# High-throughput drug screening in advanced pre-clinical 3D melanoma models identifies potential first-line therapies for NRAS-mutated melanoma

**DOI:** 10.1186/s13046-025-03539-9

**Published:** 2025-10-01

**Authors:** Cristian Angeli, Demetra Philippidou, Eliane Klein, Christiane Margue, Sagarika Ghosh, Maria Lorena Cordero Maldonado, Natascia Tiso, Giovanni Risato, Fizza Irfan, Bruno Santos, Meritxell Cutrona, Joanna Patrycja Wroblewska, Stephanie Kreis

**Affiliations:** 1 Department of Life Sciences and Medicine, University of Luxembourg, Belvaux, L-4367 Luxembourg; 2 Luxembourg Centre for System Biomedicine, University of Luxembourg, Belvaux, L-4367 Luxembourg; 3 Department of Biology, University of Padova, Via Ugo Bassi 58/B, Padova, I-35131 Italy; 4 Organoid Core Facility, Gustave Roussy, Villejuif, 94800 France

## Abstract

**Background:**

Despite significant advances in targeted (BRAFi + MEKi) and immune (anti-PD1/PD-L1, anti-CTLA4, and anti-LAG3) therapies, treatment options for NRAS^mut^ melanoma remain limited. Currently, NRAS^mut^ patients rely on immune checkpoint inhibitors, classical chemotherapy, and off-label MEK inhibitors, with over 50% experiencing rapid disease progression. One of the key challenges in developing effective targeted therapies is the lack of preclinical models that accurately recapitulate the tumor microenvironment (TME) and the intrinsic resistance of melanoma cells bearing NRAS mutations.

**Methods:**

To address this, we performed high-throughput screening (HTS) of over 1,300 compounds in 3D NRAS^mut^ melanoma spheroids. A multi-step analysis was performed to identify hits, which were further tested by performing drug-response curve (DRC) analysis. Most promising compounds were further validated using mono- and co-culture 3D in vitro models that mimic three main metastatic sites in melanoma, such as skin/dermal, lung, and liver, utilizing spheroid and hydrogel systems. Ultimately, validation was conducted using zebrafish xenograft models to enable a more refined and accurate assessment of drug response.

**Results:**

High-throughput drug screening of NRAS^mut^ melanoma spheroids identified 17 candidate compounds, which were subsequently validated through DRC analyses. Among the most promising drugs, Daunorubicin HCl (DH) and Pyrvinium Pamoate (PP) were selected for further investigation, demonstrating potent anti-melanoma activity in advanced 3D co-culture systems and zebrafish xenograft models. Notably, PP demonstrated higher cytotoxicity compared to Trametinib, the off-label MEK inhibitor, with an inhibitory effect on AKT and invasive behavior in the patient-derived metastatic melanoma cell lines. Additionally, combinatorial treatment with Trametinib resulted in additive effects on cell proliferation and viability. Importantly, both compounds showed similar efficacy in NRAS^mut^ and BRAF^wt^/NRAS^wt^ melanoma cell lines that were resistant to Trametinib (MEK inhibitor).

**Conclusions:**

Using advanced 3D melanoma models that incorporate key TME elements and zebrafish xenograft models, this study highlights the potential of Daunorubicin HCl and Pyrvinium Pamoate as novel first-line therapies for NRAS^mut^ melanoma, with a noteworthy effect also on MEKi-resistant cells. These findings support drug repurposing strategies and underscore the importance of physiologically relevant preclinical models in identifying effective therapies.

**Supplementary Information:**

The online version contains supplementary material available at 10.1186/s13046-025-03539-9.

## Introduction

Cutaneous melanoma is an aggressive cancer with rising incidence rates [1]. Its progression is largely driven by MAPK pathway activation through mutations in BRAF (~ 50%) and NRAS (~ 25%) [2, 3]. While BRAF^mut^ melanoma patients benefit from BRAFi/MEKi and immune-checkpoint inhibitor (ICIs) therapies [4, 5], targeting NRAS^mut^ remains challenging [6]. Current strategies focus on MAPK inhibition or alternative pathways but have limited success. ICIs are the first-line treatment for NRAS^mut^ melanoma [7], yet response rates are poorer than in BRAF^mut^ patients [8]. With no approved targeted therapies, novel treatments are urgently needed.

While cost-effective and straightforward in high-throughput screening (HTS) campaigns, two-dimensional (2D) cell culture models lack the complexity of in vivo tissues or tumors, such as complex architecture and cell-extracellular matrix (ECM) interactions, nutrient and waste exchange, or the O_2_-CO_2_ gradient among others. These features are present in 3D culture systems such as spheroids, which offer a more accurate representation of tissue architecture and cell interactions, facilitating a more physiologically relevant assessment of potential therapeutic compounds [9, 10]. We have recently developed multicomponent 3D melanoma models for preclinical drug testing [11]. Drug discovery via HTS traditionally requires a multi-phase process involving specialized expertise, advanced technology such as lab automation, and substantial time and economic investments, as large numbers of compounds need to be analyzed. Drug repurposing, which involves identifying new therapeutic avenues for existing or investigational drugs beyond their original indication, offers an interesting alternative to the identification of de-novo drugs, a process that is time-consuming and expensive [12, 13]. This approach reduces the risk of safety-related failures, as these drugs have already undergone safety trials, thereby potentially shortening the time required for approval.

In the present study, we applied a drug repurposing approach to conduct HTS on NRAS^mut^ melanoma cells cultured as 3D spheroids, leading to the identification of two highly effective compounds: Daunorubicin HCl and Pyrvinium Pamoate. Additionally, we evaluated the combination of these compounds with the MEKi Trametinib used off-label for NRAS^mut^ patients. Both monotherapy and combination treatments were tested in advanced pre-clinical models, including *in vitro* 3D melanoma co-cultures and in vivo zebrafish models showing promising effects of the repurposed compounds for the treatment of NRAS^mut^ melanoma patients.

## Materials and methods

### Cells and reagents

NRAS^mut^ human melanoma cell lines SKmel147 (Prof. Dr. Jochen Utikal, University Medical Center Mannheim, Germany), SKmel30 and MelJuso (DSMZ, Leibniz Institut, Germany), and the BRAF^wt^/NRAS^wt^ human melanoma cell line WM3918 (Rockland, USA) were cultured in RPMI 1640 enriched with GlutaMAX (Gibco Thermo Fisher Scientific, USA), supplemented with 10% FCS (Fetal Calf Serum, Gibco Thermo Fisher Scientific, USA) and 0.1 mg/mL Normocin (InvivoGen, USA). Patient-derived human melanoma cell lines M160915 and M161022 (Prof. Mitchell Levesque, University Hospital Zurich , Switzerland) were cultured in RPMI 1640 (Gibco Thermo Fisher Scientific, USA), supplemented with 10% FCS, 1mM Sodium Pyruvate (Gibco Thermo Fisher Scientific, USA), 4mM L-Glutamine (Gibco Thermo Fisher Scientific, USA), and 0.1 mg/mL Normocin. NHDF (normal human dermal fibroblasts) (Promocell, C-12300), MRC-5 (human lung fibroblasts) (ATCC, CCL-171), and LX-2 cells (human hepatic stellate cells) (Merk, SCC064) were cultured in DMEM enriched with GlutaMAX (Gibco Thermo Fisher Scientific, USA), supplemented with 10% FCS, 2.5% HEPES buffer 1 M (Gibco Thermo Fisher Scientific, USA), and 0.1 mg/mL Normocin. HMEC-1 (human endothelial cells) (ATCC, CRL-3243) were cultured in MCDB131 (Gibco Thermo Fisher Scientific, USA), supplemented with 10% FCS, 1 µg/mL Hydrocortisone (Sigma-Aldrich, USA), 10mM L-Glutamine, 0.1 mg/mL Normocin, and 10 ng/mL recombinant human EGF (PeproTech, USA). Trametinib (MEKi)-resistant SKmel30 and WM3918 cell lines were generated by continuous drug exposure of parental drug-sensitive cell lines to 5xIC50 and 1xIC50 concentrations, respectively, for approximately 3 months. The Binimetinib (MEKi)-resistant MelJuso cell line was generated by continuous drug exposure of the parental drug-sensitive cell line to 10xIC50 concentration of Binimetinib. All cell lines were transduced with Multiplicity of Infection (MOI) 3 of lentiviral vectors carrying reporter genes, for stable fluorescent protein expression. SKmel147, SKmel30, and M161022 were transduced with rLV.EF1.mCherry-9; NHDF, MRC-5 and LX-2 were transduced with pLenti-C-mGFP-P2A-Puro.; HMEC-1 were transduced with pLV-Bsd-CMV > tagBFP. After transduction, cells were subjected to antibiotic selection (either Puromycin or Blasticidin) and FACS-sorted using a BD FACSMelody™ Cell Sorter (BD Bioscences, USA). Cell growth was maintained at 37 °C in a humidified atmosphere comprising 5% CO2. All cell lines were regularly examined for mycoplasma contamination. Cell Line authentication was performed at Luxgen (Luxembourg).

The compound library Prestwick Chemical library^®^ (PCL, Prestwick Chemicals, USA) is composed of 1267 mainly FDA-approved compounds supplied at 10mM concentration in DMSO. The in-house “Melanoma drug library” (MDL) was generated based on literature for their effect on the different melanoma genomic subtypes. It is composed of 61 compounds supplied at 10 mM concentration in DMSO, purchased from Selleckchem. Selected hit drugs were purchased individually from Prestwick Chemicals and dispensed in a specific ready-to-use source plate. For cell treatments outside the HTS workflow: Trametinib (#S2673), Daunorubicin HCl (#S3035), and Pyrvinium Pamoate (#S5816) were purchased from Selleckchem (Germany). Staurosporine (#CAYM81590-1) was purchased from Cayman Chemical (USA).

### 3D high-throughput screening

HTS assays were performed using the HTS platform, “Disease Modelling and Screening Platform” (DMSP) of LIH/LCSB, Luxembourg. The platform is equipped with two liquid handler workstations (Biomek NXp and Biomek FXp; Beckman Coulter), two integrated incubators (Cytomat 24-C; Thermofisher), an acoustic droplet ejector (Echo 550; Labcyte), a multimode plate reader (SpectraMax i3;Molecular Devices), a confocal high-content microscope (CV8000; Yokogawa) equipped with solid lasers (wavelengths: 405/488/561 nm) and emission filters (445/45 nm, 525/50 nm, 600/37 nm), and an integrated robotic arm on rail (SCARA; Beckman Coulter). Cells were seeded in 384-well U-bottom ULA black plates (Corning^®^, 4516, USA) at a density of 5 × 10^3^ cells/well in 20 µL/well, centrifuged at 500 x g for 5 min, and incubated for 72 h at 37 °C and 5% CO_2_ to allow for spheroid formation. After the compounds from the PCL and MDL libraries were dispensed (one compound per well) at nanoliter range using the acoustic droplet ejector, a further 40 µL of fresh culture medium were added and spheroids were incubated for 5 days at 37 °C and 5% CO_2_. Every compound was dispensed at a final concentration of either 1 µM or 10 µM, each of them in duplicate (on two separate plates), with a final DMSO concentration of 0.1%. Side wells were dedicated to a pre-selected positive control compound for the screening (Foretinib 30 µM) and negative controls (DMSO 0.1%), and the first and last rows and columns of the plate were excluded to reduce edge effects. Additional plates were added to the screen with the compounds falling into the edge effect area. To detect and quantify the spheroid response to drugs we extracted the maximum intensity projection (MIP) area for each spheroid by applying high-content image analysis (see below). The MIPs were obtained from the Calcein AM (Cayman Chemical, USA) signal, thus representing a surrogate measure of cell viability, and informing on the size or growth of spheroids exposed to the drugs. At the end of the drug treatment, Calcein AM was added 4 X concentrated as 20 µL/well to reach a final concentration of 4 µM (80 µL final volume in each well) and incubated for 2 h at 37 °C. We initially used different Calcein AM concentrations and incubation times to optimize the ratio signal-to-noise to give us the most robust signal for imaging of spheroids. Confocal images were acquired using a 10x objective, 488 nm laser 525/50 nm emission filter, Z-stack acquisition (e.g. the Z-stack consisted of 40 slices taken sequentially with 10 μm step size for a total span of 390 μm) and on-the-fly generation of MIP images mode. A mock test was run before each HTS campaign to check the quality of the cells and assay, following the same seeding and timing procedures and including a drug response performed using a 3-fold dilution series of Foretinib starting at 10 µM.

### Hit drug identification

CellPathFinder^®^ was used to analyze MIP images and extract the total spheroid area in each well. In brief, a segmentation mask was created on the Calcein AM green-fluorescent signal, which allowed for the calculation of the radius and area of the spheroid MIP. The software summed all the area’s segments outputting a total Calcein AM area per well (µm^2^). The application of a statistical test (Grubb’s test), followed by visual inspection, removed outliers (such as failure of segmentation) from the set of data. The Z’-factor was calculated for each plate of the primary screening as a quality control step. The raw MIP measures were used to mathematically set a plate-specific cut-off for determining hit drugs, by applying the following formula: averageDMSO − (3 x standard_deviationDMSO) [14]. Drugs were taken into consideration if the raw MIP area values were below the cut-off in both duplicate plates. Data was normalized for the corresponding DMSO controls in the plate and expressed as percentage of residual MIP. Only drugs below 50% of this residual MIP in both duplicates from the set of values below the acceptable SD cut-off, were processed into the final selection step. Finally, we visually examined the MIP images to confirm the drug’s effect and rule out false positives. Additionally, we applied extra criteria, such as reviewing existing literature, to compile a final list of effective drugs. Rstudio was used for the analysis and the creation of relative plots.

### Drug-response curve analysis in HTS fashion for hit validation

Drug-response curves (DRC) to determine the relative half-maximal inhibitory concentration (IC50) values were generated for the 17 selected hits using the same approach as described for the primary screening. Drugs, including the positive control Foretinib, were dispensed in duplicate using a 3-fold dilution series from the dedicated source plate, starting from 10µM with 10 dilutions. Cell viability was assessed using Calcein AM (as previously described). Data were normalized by the DMSO control within each plate. GraphPad 10.3.1 software (GraphPad, USA) software and non-linear regression (four parameters) analysis were used to extrapolate IC50 and R^2^ values for each tested compound.

### 3D mono- and multi-component spheroid generation

Mono-component spheroids were generated in 384-well ULA U-bottom plates (S-Bio^®^, MS-9384UZ, Japan) as follows: melanoma cells were seeded at a density of 0.5-1 × 10^3^ cells/well in 80 µL of RPMI. The plate was centrifuged 500 *x g* for 5 min and incubated at 37 °C and 5% CO_2_ for 96 h.

Multi-component spheroids were generated as described before [11]. Melanoma cells, fibroblasts or hepatic stellate cells, and endothelial cells were seeded at a cellular ratio of 1:3:3 in 384-well black/clear round bottom ultra-low attachment spheroid microplates (Corning^®^, 4516, USA). Melanoma cells and HMEC-1 were seeded together at densities of 0.5 × 10^3^ cells/well and 1.5 × 10^3^ cells/well, respectively, in 40 µL of RPMI. The plate was centrifuged 500 *x g* for 5 min and incubated. After 24 h of incubation, either NHDF, MRC-5, or LX-2 were seeded at densities of 1.5 × 10^3^ cells/well in a further 40 µL of RPMI, on top of the preformed spheroids, the plate was then centrifuged 500 *x g* for 5 min and incubated at 37 °C and 5% CO_2_ for 72 h.

### 2D and 3D DRC and IC50 determination

Generation of DRCs and determination of IC50 values of drugs in 2D tested in non-cancerous cells (NHDF, MRC-5, LX-2, and HMEC-1) were performed as follows: cells were seeded in a 96-well black plate (µClear Greiner^®^, Belgium) at a density of 5 × 10^3^ cells/well in 100 µL of cell line-specific medium. Drugs were diluted in a 3-fold dilution series for 8 dilutions, with starting concentrations of Daunorubicin HCl and Pyrvinium Pamoate of 10 µM. Cell viability was determined with the CellTiter-Glo^®^ 3D Cell Viability Assay (Promega, USA). Upon 5 days of treatment, a microplate reader Cytation 5 Cell Imaging Multi-Mode Reader (Agilent BioTek, USA) was used for luminescence measurements. The IC50 experiments were performed in technical and biological triplicates. Dose-response curves and IC50 values were generated with GraphPad 10.3.1 software (GraphPad, USA) and determined with the non-linear log (inhibitor) vs. response-variable slope (four parameters) equation. For selected melanoma cells the determination of IC50 values of drugs tested was performed in 3D as follows: cells were seeded in 384-well U-bottom ULA plates (S-Bio^®^, MS-9384UZ, Japan) at densities of 0.5-1 × 10^3^ cells/well in 80 µL/well, centrifuged at 500 *x g* for 5 min, and incubated for 4 days at 37 °C and 5% CO_2_. Drugs were diluted in a 3-fold dilution series for 10 dilutions, with starting concentrations of Daunorubicin HCl of 10 µM and Pyrvinium Pamoate of 1 µM. Before drug and cell viability reagent were added, spheroids were visually inspected utilizing a bench-top microscope as a quality control step. After 5 days of treatment, cell viability was determined with the CellTiter-Glo^®^ 3D Cell Viability Assay (Promega, USA). A microplate reader Cytation 5 Cell Imaging Multi-Mode Reader (Agilent BioTek, USA) was used for luminescence measurements. The IC50 experiments were performed in technical and biological triplicates. Dose-response curves and IC50 values were generated with GraphPad 10.3.1 software (GraphPad, USA) and determined with the non-linear log (inhibitor) vs. response-variable slope (four parameters) equation.

### 3D synergy assay

SKmel30 and SKmel147 cells were seeded at a density of 0.5 × 10^3^ cells/well in 384-well ULA plates (S-Bio^®^, MS-9384UZ, Japan) and spheres were allowed to form for 4 days before addition of drugs. They were treated for 5 days with either single drugs or combinations of Trametinib and either Pyrvinium Pamoate or Daunorubicin HCl in a matrix format at a fixed 1:2 dilution range. Drug concentrations were pre-determined based on each inhibitor’s IC50 value. Cell viability was assessed with the CellTiter-Glo^®^ 3D Cell Viability Assay (Promega, USA). Synergy scoring was determined using the “inhibition readout” (calculated as “100 - Cell Viability”) of the online SynergyFinder software version 3.0 (https://synergyfinder.fimm.fi) and implementing the ZIP calculation method, as published before [15]. Zero Interaction Potency (ZIP) scores < − 10 and > 10 correspond to antagonist and synergistic effects, respectively.

### 3D proliferation kinetic and end-point assay

Kinetic (time-lapse microscopy) cell proliferation and endpoint cell viability, under drug treatments, were evaluated as described before [11]. In brief, either mono- or -multicomponent spheroids were generated as previously described using labeled cells to allow the tracking of the different cell types. After spheroid generation, 40 µL medium were removed from each well and replaced with 40 µL medium supplemented with 2 times concentrated compounds and controls. The plate was centrifuged at 500 x g for 5 min and placed in an incubator (BioSpa8, Agilent BioTek, USA) connected to an automated live-cell imaging system (Cytation 10, Agilent BioTek, USA). Images were acquired every 12 h for 5 days using a 10x magnification objective and 590 nm LED and a Texas Red filter cube (Excitation 586/15 nm, Emission 647/57 nm) to track melanoma fluorescence signal over time. On day 5, spheroid cell viability was determined using the CellTiter-Glo^®^ 3D Cell Viability Assay (Promega, USA). A microplate reader Cytation 5 Cell Imaging Multi-Mode Reader (Agilent BioTek, USA) was used for luminescence measurements. Kinetic and end-point cell proliferation data were analyzed and plotted with GraphPad 10.3.1 software (GraphPad, USA).

### Confocal microscopy of 3D multi-component spheroids

Confocal images of 3D multi-component spheroids were acquired using the Cytation 10 (Agilent BioTek, USA) confocal microscope with spinning disk technology. The instrument is equipped with a laser combiner (spectral range 398–643 nm) and a DAPI filter cube (Excitation 390/40 nm, Emission 442/42 nm), a GFP filter cube (Excitation 472/ 30 nm, Emission 520/35 nm), and a TRITC filter cube (Excitation 556/20 nm, Emission 600/37 nm). Pictures were acquired using a 20x magnification objective.

### 3D apoptosis and cell death assays using confocal microscopy

Melanoma cells were seeded in 384-well black U-bottom ULA microplates (Corning^®^, USA) at densities of 0.5 × 10^3^ cells/well in 80 µL/well of medium, centrifuged at 500 x g for 5 min, and incubated for 2 days at 37 °C and 5% CO2. Upon removal of 40 µL/well of medium, drugs were dispensed 2 times concentrated in 40 µL/well of medium, centrifuged at 500 x g for 5 min, and incubated for 5 days at 37 °C and 5% CO2. The positive control, Staurosporine at 1µM concentration was added 24 h previous to the end of the assay, for strong induction of apoptosis and cell death. CellEvent™ Caspase-3/7 Detection Reagent (Invitrogen, Thermo, USA) and SYTOX™ Blue Dead Cell Stain (Invitrogen, Thermo, USA) were added and incubated at 37 °C for at least 2 h. Cytation 10 was used to acquire multiple images in z-stacking using DAPI, GFP, and TRITC filter cubes and a 20x magnification objective. Brightfield pictures were also acquired at 20x magnification. Maximum intensity projected (MIP) images were generated using Gen5 (Agilent BioTek, USA). For mCherry-expressing melanoma cell lines, the mCherry signal was used to visualize the total spheroid mass, while for non-labeled melanoma cells, brightfield images were used to visualize the total spheroid mass.

### 3D invasion assay

Melanoma cell lines SKmel147 and M160915 were seeded in ultra-low attachment BIOFLOAT™ 96-well plates (Facellitate, Germany) in densities of 2,5 × 10^3^ and 5 × 10^3^, respectively. After 3 days of spheroid formation, they were embedded between two layers of Collagen type I, containing 2 mg/ml Collagen type I (MercMillipore, Germany), 1% FCS (Gibco Thermo Fisher Scientific, Waltham, USA) in RPMI (Gibco Thermo Fisher Scientific, USA). The pH of the collagen solution was adjusted to 7.4 using 1 M NaOH. 50 µl per well of Collagen I solution was pipetted into an optically clear, black-walled 96-well plate (µClear Greiner^®^, Belgium) and left to polymerize for 5 min at 37 °C. Next, one spheroid per well was transferred on top of the collagen layer and immediately covered with 50 µL of collagen solution and polymerized for 15 min at 37 °C. Next, 100 µl of medium containing either 0,5% DMSO (negative control) or 2 times IC50 concentration of the drug was added on top of the collagen layer. For each experimental condition, 8 spheroids were used. Pictures were taken on day 0 (immediately after embedding) and after 3 days of collagen embedding, using Cytation 10 (Agilent BioTek, USA) manual imaging mode and 4x magnification. The area of cellular invasion was analyzed using ImageJ software (Fiji). Statistical analysis was performed using GraphPad 10.3.1 software (GraphPad, USA).

### Western blot analysis

Cells were seeded in 6-well Aggrewell plates (StemCell, USA) at densities of 0.5-1 × 10^3^ cells/well in 5 mL of medium, centrifuged at 100 x g for 5 min, and incubated for 4 days. Drugs were dispensed and cells were incubated for 3 and 5 days. Cell lysis was performed on ice with cold lysis buffer (RIPA 1X containing cOmplete phosphatase inhibitor, Roche, Switzerland), protein concentration was determined using Pierce™ BCA Protein Assay Kit (Thermo, USA), and protein lysates were further analyzed by SDS-PAGE and Western Blot. The detection of enhanced chemiluminescence signals was performed as previously described [16]. Primary antibodies used in the study were: GAPDH (1:5000, polyclonal, #G9545, Rabbit, Sigma, USA), ERK (1:1000, Rabbit, L34F12, #CST4696S, CellSignaling, USA), pERK (1:1000, Rabbit, D13.14.4E, #CST4370S, CellSignaling, USA), AKT (1:1000, Mouse, 4OD4, #CST2920S, CellSignaling, USA), pAKT (Ser473) (1:1000, Rabbit, D9E, #CST4060S, CellSignaling, USA), PRAS40 (1:1000, Rabbit, #CST2610, CellSignaling, USA), pPRAS40 (1:1000, Rabbit, C77D7, #CST2997, CellSignaling, USA), S6 (1:1000, Rabbit, 5G10, #CST2217, CellSignaling, USA), pS6 (1:1000, Rabbit, 91B2, #CST4857, CellSignaling, USA), p70S6K (1:1000, Rabbit, 49D7, #CST2708, CellSignaling, USA), pp70S6K (1:1000, Rabbit, 108D2, #CST9234, CellSignaling, USA), 4EBP1 (1:1000, Rabbit, 53H11, #CST9644, CellSignaling, USA), p4EBP1 (1:1000, Rabbit, 236B4, #CST2855, CellSignaling, USA). All primary and HRP-conjugated secondary antibodies were purchased from Cell Signaling Technology (Boston, USA).

### Immunofluorescence staining

Cells were seeded in multi-chamber slides (Ibidi, Germany) at a density of 20 × 10^3^ cells/chamber in 300 µL of medium/chamber and incubated at 37 °C and 5% CO2 overnight. Drugs were dispensed and incubated for 72 h. Cells were fixed in 4% PFA for 10 min at RT and permeabilized with 0.3% Triton X solution for 10 min at RT. Afterward, cells were washed with PBS and incubated in a blocking solution (10% FCS) for 1 h. Primary antibodies were added and incubated at 4 °C overnight. β-Catenin (1:100, Rabbit, #8480S, Cell Signalling^®^) and γH2AX (1:200, Mouse, #80312, Cell Signalling^®^). Secondary antibodies conjugated with a fluorophore Alexa Fluor 488 (goat anti-rabbit, 1:500, Thermo), Alexa Fluor 647 (donkey anti-rabbit, 1:500, Thermo), Phalloidin Alexa 647 (Thermo), and DAPI (1:1000) were added and incubated for 1 h at RT. Confocal images were acquired using Cytation 10 with a 60x objective, and with DAPI, GFP and CY5 filter cubes.

### Hydrogel-embedded melanoma co-culture

Melanoma-TME hydrogel encapsulation co-cultures were generated using transglutaminase cross-linkable poly (ethylene glycol) (PEG) hydrogels previously described [17]. A ready-to-use kit consisting of frozen aliquots of the 3% PEG precursor solution (8-arm 40 kDa PEG macromers bioconjugate with RGD adhesion and MMP-cleavable peptide motives) and of the activated Human Factor XIII (FXIIIa) were purchased (Ectica Technologies, Switzerland). The cell suspension was created by mixing mCherry-expressing melanoma cells at a density of 2–4 × 10^4^/100µL with HMEC-1 expressing BFP at a density of 20 × 10^4^/100µL, and with either NHDF, or MRC-5, or LX-2 expressing GFP at a density of 20 × 10^4^/100µL, centrifuged at 300 x g for 3 min and supernatant was removed, and 45 µL of complete RPMI were added. Afterwards, 43 µL of PEG precursor solutions were added and gently mixed to dissolve the cellular pellet. Then, 12 µL of FXIIIa was added, and the solution was gently mixed without introducing bubbles. 5 µL of solution was dispensed in each well in a black 96-well plate (µClear Greiner^®^, Belgium) to create homogeneous domes and incubated at RT for 5 min until polymerization. 200 µL/well of RPMI supplemented with 10ng/mL of VEGF (Peprotech, USA) was dispensed in each well and incubated for 3 days at 37 °C and 5% CO2. 2X concentrated drugs were added in 100µL/well of fresh medium, upon removal of 100 µL/well of the old medium, and further incubated for 5 days at 37 °C and 5% CO2. The confocal microscope Cytation 10 (Agilent, BioTek, USA) was used to acquire multiple images in z-stack modality using DAPI, GFP, and TRITC filter cubes and a 20x magnification objective, selecting 4 ROIs per well. Maximum intensity projected images were analyzed using ImageJ (Fiji).

### Zebrafish husbandry, determination of maximum tolerated concentrations of drugs, and xenografts

Zebrafish experiments were performed in two different institutions, the Zebrafish Facility of the University of Padova (under Italian Ministry of Health Authorization n. 1111/2024-PR (OPBA prot. D2784.185)) and the Aquatic Platform of the Luxembourg Centre for Systems Biomedicine at the University of Luxembourg (RRID: SCR_025429), in collaboration with Professor Natascia Tiso and Dr. Maria Lorena Cordero-Maldonado, respectively. Adult *nacre* zebrafish lines were housed in each facility according to standard protocols [18, 19]. Embryos were obtained by natural spawning and reared until the experiments at 2 dpf in E3 medium at 28° C. First, to determine the maximum tolerated concentration (MTC) of the drugs to be tested in the xenografts (Trametinib, DH and PP), we first treated non-injected naïve 2 dpf *nacre* larvae with serial dilutions of drugs of interest to determine the highest tolerated and non-toxic concentration until 5 dpf. Larvae viability and development were monitored daily during drug treatment. The cut-off of 20% mortality and no developmental defect was set to determine the MTC. Second, for the performance of the cell transplantations, on the day of the injections, the 2 dpf embryos were manually dechorionated and anesthetized with buffered tricaine (80 mg/l, Sigma-Aldrich). SKmel147-mCherry and MelJuso-RES-mCherry cell lines were detached using phenol red-free TryplE reagent (Gibco Thermo Fisher Scientific) and resuspended in PBS at a concentration of 2 × 10^5^cells/ µL. The cells were injected into the yolk as a single droplet (around 100 cells per embryo) using a World Precision Instrument (Sarasota, USA) or FemtoJet 4X (Eppendorf, Germany) microinjectors. PBS with phenol red was injected as a vehicle control. After 24 h, the larvae were fluorescently assessed for successful cell implantation and subjected to drug treatment with 12 nM Trametinib, 1 µM DH, 111 nM of PP, and their combinations for 3 days at 32 °C. Larvae viability was monitored daily. After 3 days, larvae were anesthetized as described above, and photos of xenografts were taken using an M165 FC microscope with DFC7000T camera (Leica Camera, Germany) or Nikon SMZ25 fluorescent stereomicroscope (Nikon Instruments, Japan). Data was analyzed based on fluorescence intensity to measure xenograft area and number of cells using the “Measurements” tool of the Volocity 6.0 software (Perkin Elmer, Italy).

### Statistical analysis

All experiments represent at least 3 biological replicates. Statistical analysis was performed using GraphPad 10.3.1 software (GraphPad, USA). The Gaussian distribution of data was assessed with Shapiro-Wilk normality test. Data following Gaussian distribution was analyzed using Ordinary one-way ANOVA with Dunett’s multiple comparison test. Data not following Gaussian distribution was analyzed using ordinary Kruskal-Wallis with Dunn’s multiple comparison test. One sample t-test was used to analyze data expressed as a percentage of the untreated control (normalized to 100%).

## Results

### High-throughput drug screening and dose-response assays identify promising novel compounds for NRAS^mut^ melanoma

**Fig. 1:**
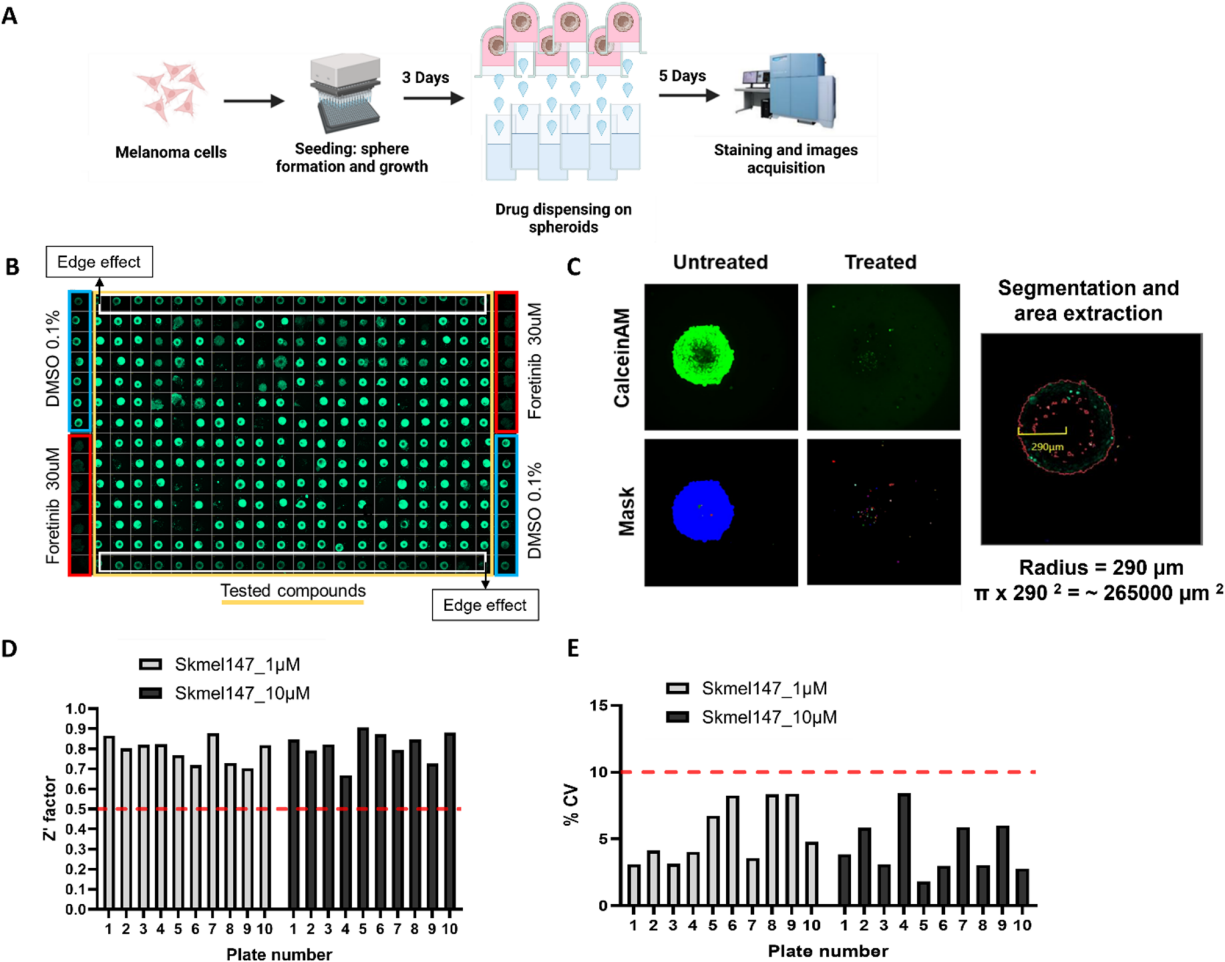
High-Throughput Screening (HTS) allows the testing of hundreds of compounds on NRAS^mut^ melanoma 3D spheroids. **A**) Representation of the HTS workflow on SKmel147 melanoma spheroids. Melanoma cells were cultured in 384-well U-bottom ULA plates for 3 days. Drugs were dispensed using an acoustic liquid handler. After 5 days of treatment, staining and image acquisition were performed. **B**) Representative plate layout of HTS. The yellow box highlights the wells where drugs were dispensed; white boxes highlight the wells affected by the edge effect; blue and red boxes highlight the wells dedicated to DMSO 0.1% (vehicle/negative control) and Foretinib at 30 µM (positive control), respectively. **C**) Representative pictures of residual maximum intensity projection (MIP) used for segmentation and mask generation eliciting the Calcein AM fluorescent signal during high-content imaging analysis, and subsequent total spheroid area calculation. **D**) Z’-factors for each screened plate. A cut-off was set at 0.5. Libraries were distributed on five plates, with 2 replicates per drug. Plates 1 to 5 represent the first replicate, and plates 6 to 10 represent the second replicate of tested drugs. E) A cut-off of coefficients of variation (%) was set to 10% for each screened plate.

We evaluated the effects of two drug libraries, the commercial Prestwick Chemical Library^®^ (1267 compounds) and an in-house Melanoma Drug Library (61 compounds) selected based on literature and previous data, on SKmel147 NRAS^mut^ melanoma cells cultured as 3D spheroids. Drugs were tested at 10 µM and 1 µM to minimize off-target effects. A HTS workflow was developed using a fully automated platform (Fig. 1A), with individual drugs dispensed in a specific plate layout (Fig. 1B). The negative control was 0.1% DMSO, while Foretinib at a concentration of 30 µM was used as the positive control for inducing cell death. To account for the edge effect, additional plates were included to test drugs dispensed onto spheroids located in edge effect-affected wells. Cell viability was evaluated using Calcein AM staining, followed by signal-based spheroid area segmentation, and area analysis (Fig. 1C), in accordance with the standard protocol implemented by the DMSP facility.

Standard quality control (QC) parameters, including Z’-factor (> 0.5) (Fig. 1D) and coefficient of variation (< 10%) (Fig. 1E), were assessed across all plates, ensuring robust data for hit identification along our HTS campaign.

**Fig. 2:**
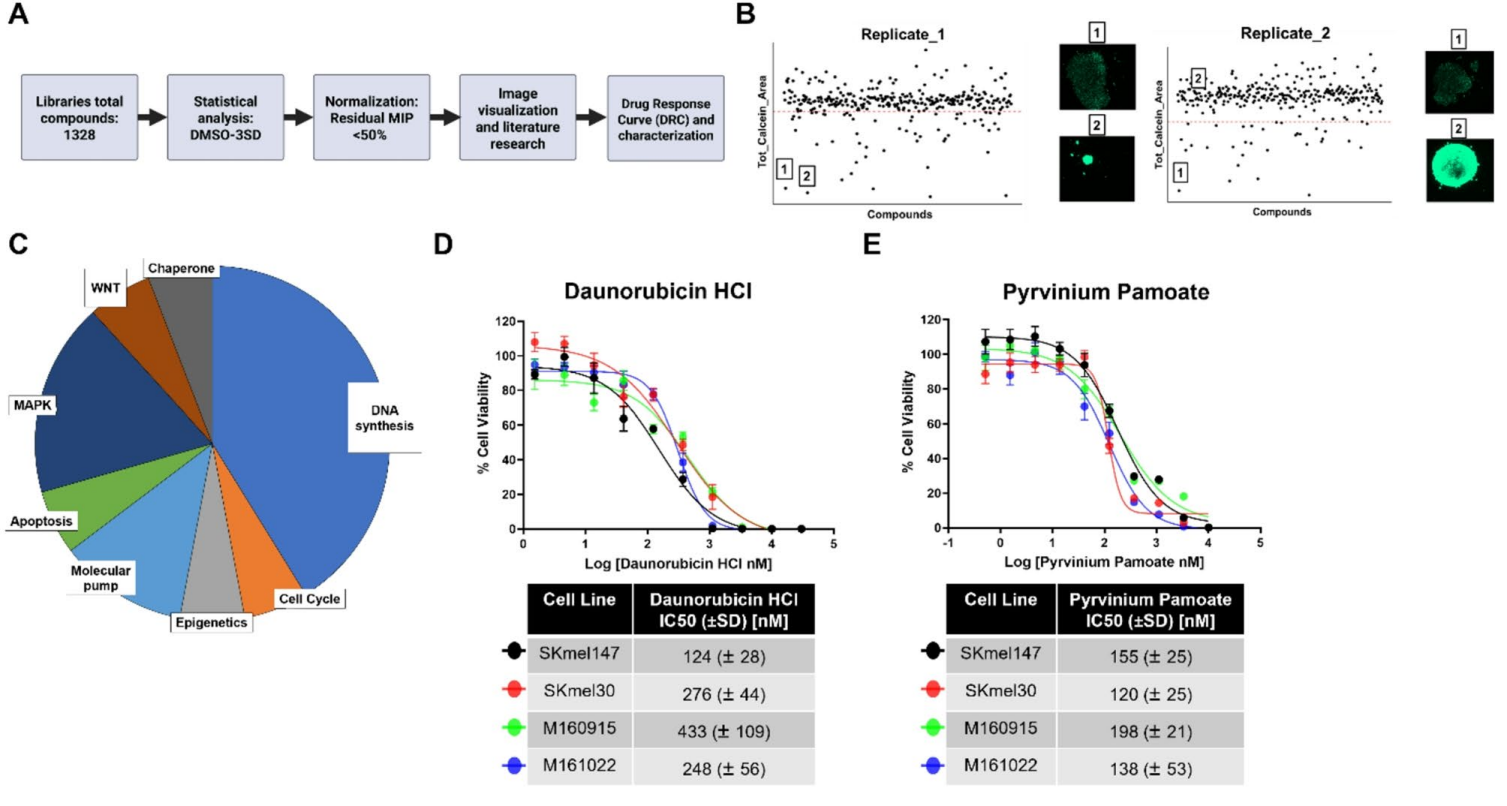
A multistep selection process identifies novel compounds for potential treatment of NRAS^mut^ melanoma. (**A**) Schematic representation of the multistep process of drug selection for subsequent drug-response curve (DRC) validation. DMSO-3STD: deviation of at least 3 standard deviations from the mean of DMSO control. (**B**) Scatter plots represent the total MIP Calcein AM area of a series of compounds screened. The left panel represents the first replicate, and the right panel represents the second replicate. The red dashed line indicates the plate-specific cut-off: only compounds that were below the cut-off in both replicates were selected (compound 1 here). Next to the scatter plots: confocal images of melanoma spheroids treated with either compound 1 or 2 in both replicates. (**C**) The pie chart represents the pathways targeted by the 17 selected hit compounds. **D** and **E**) Drug-response curves of Daunorubicin HCl and Pyrvinium Pamoate generated on four NRAS^mut^ melanoma cell lines (SKmel147, SKmel30, M160915 and M161022), utilizing CellTiter-Glo^®^ 3D Cell Viability Assay as readout after 5 days of treatment. Reported IC50 values in the tables are mean (± SD) of 3 independent biological replicates.

Hit identification involved multiple steps (Fig. 2A). First, a statistical model analyzed the Maximum Intensity Projection (MIP) spheroid area values that were obtained by automated image segmentation using the Calcein AM signal. Only the measurements that deviated by at least three standard deviations from the mean of the DMSO control (cut-off) [14] in both duplicates were considered for the next selection step (see Materials & Methods for details) (Fig. 2B).

Next, all spheroid MIP data were normalized to the average MIP measured in spheroids treated with DMSO. The hits were selected based on values below 50% of residual MIP, following exposure to drugs. The spheroid shrinkage effects were confirmed by manual visual inspection, and additional information, such as FDA status and pathway relevance, was reviewed.

Seventeen promising drugs (Table 1) were selected based on well-established roles in targeting pathways critical to melanoma progression (Fig. 2C), including DNA synthesis and damage, epigenetic regulation, and apoptosis.

**Table 1:** Important features of the 17 selected hit drugs. Target, FDA status, therapeutic effect and targeted pathway. Highlighted in bold are Daunorubicin HCl and Pyrvinium Pamoate , subsequently selected for further validations

Compound	Target	FDA approved	Therapeutic effect	Targeted pathway
AZD6738	ATR	NO	Antineoplastic	DNA synthesis
Camptothecine	Topoisomerase I	NO	Antineoplastic	DNA synthesis
CHIR-124	CHK1	NO	Antineoplastic	Cell Cycle
Cladribine	Ribonucleotide reductase	YES	Antineoplastic	DNA synthesis
**Daunorubicin HCl**	Topoisomerase II	YES	Antibacterial	DNA synthesis
Entinostat	HDAC	NO	Antineoplastic	Epigenetic
Epirubicin HCl	Topoisomerase II	YES	Antineoplastic	DNA synthesis
Irinotecan HCl Trihydrate	Topoisomerase I	YES	Antineoplastic	DNA synthesis
Lanatoside C	Plasma membrane Na+/K + ATPase	NO	Cardiotonic	Molecular Pump
Obatoclax	Bcl-2	NO	Antineoplastic	Apoptosis
PD0325901	MEK	NO	Antineoplastic	MAPK
Proscillaridin A	Plasma membrane Na+/K + ATPase	NO	Antiarrhythmics	Molecular Pump
**Pyrvinium Pamoate**	CK1α	YES	Anthelmintic	WNT
TAK-733	MEK	NO	Antineoplastic	MAPK
Topotecan	Topoisomerase I	YES	Antineoplastic	DNA synthesis
Ulixertinib	ERK	NO	Antineoplastic	MAPK
XL888	HSP90	NO	Antineoplastic	Epigenetic

Notable compounds included topoisomerase inhibitors and poisons that target DNA stability (such as Daunorubicin HCl ), a checkpoint kinase 1 (CHK1) inhibitor (CHIR-124), and epigenetic modulators like Entinostat (HDAC inhibitor), for example. Additionally, heat-shock protein 90 (HSP90) and BCL-2 inhibitors (e.g. XL888 and Obatoclax) were selected alongside cardiac glycosides (such as Lanatoside C, Proscillaridin A), inhibiting the Na+/K + ATPase pump, which represent an emerging therapeutic target in cancer [20, 21]. MAPK pathway inhibitors (such as PD0325901, TAK-733, or Ulixertinib) and casein kinase 1 α (CK1α) agonist Pyrvinium Pamoate, targeting the WNT-β-Catenin pathway, were also included.

Validation of hits was performed via high-throughput dose-response curve (DRC) analysis (Supplementary Fig. 1A). DRC generation confirmed the inhibitory effects of individual drugs, displaying IC50 values ranging from 2 nM to > 1 µM, with only a few compounds unable to derive IC50 values. Compounds targeting DNA stability (such as Cladribine, Topotecan, Irinotecan, and Daunorubicin HCl) showed IC50 values below 50 nM, demonstrating strong effect of the compounds as well as indicating the sensitivity of our HTS assay. Other efficient compounds included Lanatoside C, Proscillaridin A, Pyrvinium Pamoate, XL888, and PD0325901, and generated IC50 values below 200 nM. Surprisingly, potent MAPK inhibitors (such as TAK-733 and Ulixertinib) or cell cycle inhibitors (CHIR-124) exhibited limited inhibitory effects.

Collectively, these HTS data identified promising compounds for further validation as potential candidates for the treatment of NRAS^mut^ melanoma.

### NRAS^mut^ melanoma cells are sensitive to Daunorubicin HCl and Pyrvinium Pamoate

Further investigation primarily focused on two compounds, DH and PP, as potential first-line treatments for NRAS^mut^ melanoma. In addition to their strong inhibitory effects on SKmel147 melanoma cells observed during the HTS and DRC campaign, DH was selected from the major targeted pathway category, ‘DNA synthesis’, while PP was chosen for its notable impact on the WNT-β-Catenin pathway, which plays a critical role in melanoma progression [22, 23]. Furthermore, both compounds were selected based on their FDA approval status, underscoring a drug repurposing approach, and for their largely understudied roles in melanoma and their reported interesting effects in other cancer types [24, 25]. Both drugs showed strong efficacy across four treatment-naïve melanoma cell lines cultured as spheroids (SKmel147, SKmel30, M160915) or 3D aggregates (M161022) (Fig. 2D-E).

Despite their FDA-approved status, additional testing on non-cancerous cells (Supplementary Fig. 1B-C) revealed higher IC50 values for PP compared to melanoma cells (“Dermal Fibroblasts”-NHDF = 998 nM; “Lung Fibroblasts”-MRC5 = 252.3 nM; “Hepatic Stellate Cells”-LX-2 = 209.9 nM; “Endothelial Cells”-HMEC-1 = 505.6 nM), indicating therapeutic safety. Meanwhile, LX-2 and HMEC-1 cell lines displayed sensitivity to DH compared to PP (Supplementary Fig. 1B-C). This observation is consistent with the concept of drug repurposing that, despite FDA approval and established safety profiles, certain normal cells may exhibit differential sensitivity, potentially leading to adverse effects in patients. Moreover, the increased sensitivity observed in some non-cancerous cells may be attributable to the different culture methods employed, with these cells cultured on adherent plates and melanoma cells cultured as spheroids, respectively. As expected, under our experimental conditions, these non-cancerous cells did not form spheroids (data not shown).

In conclusion, DH and PP demonstrated very good activity against NRAS^mut^ melanoma cells grown as spheroids with minimal effects on most of the non-cancerous cells, underscoring their potential for drug screening and repurposing in melanoma therapy.

### Daunorubicin HCl and Pyrvinium Pamoate inhibit proliferation and viability of NRAS^mut^ melanoma cells cultured in 3D

**Fig. 3:**
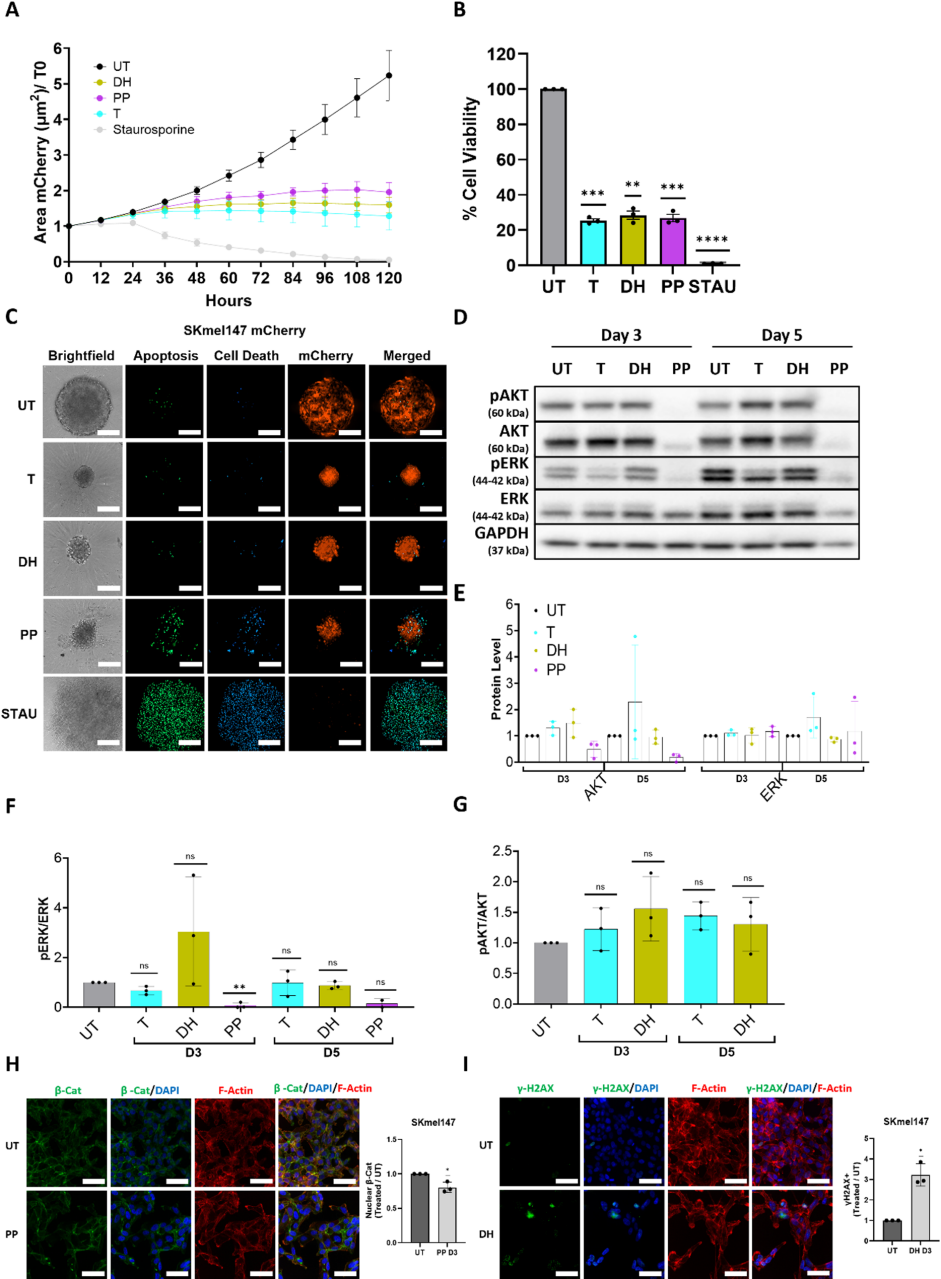
Daunorubicin HCl and Pyrvinium Pamoate induce inhibition of proliferation and viability and trigger cytotoxic effects in SKmel147 spheroids. (**A**) Proliferation of SKmel147-mCherry spheroids treated for 5 days with indicated drugs. Fluorescent images depicting the spheroid area were acquired every 12 h. (*n* = 3, mean ± SD). (**B**) Cell viability of SKmel147-mCherry spheroids was assessed after 5 days of drug treatment. Data are normalized to the untreated control. Staurosporine was used as positive control at 200 nM in A and B. (*n* = 3, mean ± SD); (**C**) Representative pictures of apoptosis and cell death detection in SKmel147-mCherry spheroids that were treated with different compounds for 5 days. Staurosporine was used as positive control at 1µM. Apoptosis (green) and cell death (blue) were measured upon the addition of the CellEvent Caspase-3/7 and Sytox Blue detection reagents, respectively. Confocal images (20x magnification) of single spheroids are shown. Scale bar = 200 μm (*n* = 3). (**D**) Western blot of whole cell lysates from SKmel147 spheroids treated for 3 and 5 days with either Daunorubicin HCl (DH), Pyrvinium Pamoate (PP) or Trametinib (T). (**E**) Quantification of the total AKT and ERK protein levels in SKmel147, normalized to GAPDH. **F-G**) pERK/ERK and pAKT/AKT ratios respectively in SKmel147 spheroids. GAPDH was used as a loading control; Data were normalized by and to day-specific untreated controls. Representative blots of three biological replicates are shown. (**H**) Immunofluorescence (IF) staining of β-Catenin (green), nuclei (DAPI, blue), and F-Actin (Phalloidin, red); Bar plot shows quantification of the relative β-Catenin nuclear signal. (**I**) IF staining of γH2AX (green), nuclei (DAPI, blue), and F-Actin (Phalloidin, red); Bar plot shows quantification of the relative γH2AX nuclear signal. Cell-line specific IC50 drug concentrations were used for spheroid stimulations in the different assays. UT: untreated, STAU: Staurosporine. One sample T-test was used in B, F, G, H and I for statistical significance testing (ns = not significant, *p ≤ 0.05, **p ≤ 0.01, ***p ≤ 0.001, ****p ≤ 0.0001).

To further elucidate the action of DH and PP on NRAS^mut^ melanoma cells, a proliferation assay was performed using time-lapse microscopy by tracking over time the proliferation of three NRAS^mut^ melanoma cell lines, cultured as spheroids and constitutively expressing the mCherry fluorescent protein. The MEKi Trametinib (T) and Staurosporine (STAU), a well-characterized apoptosis inducer, served as positive controls. The fluorescent signal emitted by melanoma cells was used to follow spheroid sizes dynamically, enabling the evaluation of the compounds’ effect on cellular proliferation. Previously determined IC50 values for DH, PP, and Trametinib were used to assess the drug efficacy across the different assays. Spheroids were treated for five days in accordance with the protocol established in the HTS assay. PP and Trametinib induced a pronounced reduction in melanoma spheroid/3D aggregate proliferation across all tested cell lines (Fig. 3A, Supplementary Fig. 2A & 3A).

DH exhibited a similar inhibitory effect in SKmel147 and M161022 cells (Fig. 3A, Supplementary Fig. 3A), however, with weaker effects on SKmel30 cells (Supplementary Fig. 2A). This reduced efficacy of DH on SKmel30 may be explained by the cell line’s unique capacity to respond to DNA damage, possibly due to a TP53 gene deletion (Cellosaurus SK-MEL-30; CVCL_0039). Although no studies have directly examined the association between TP53 deficiency in SKmel30 cells and enhanced survival following treatment with DNA-damaging agents, potentially due to a unique capacity to respond to DNA damage, recent studies have demonstrated similar findings using other TP53-deficient cell lines [26, 27]. In support of this, SKmel30 resulted in having the lowest levels of DNA damage (γH2AX) upon DH treatment. All 3 cell lines demonstrated significant reduction in cell viability of DH and PP-treated spheroids/aggregates compared to untreated controls (Fig. 3B, Supplementary Fig. 2B & 3B). Consistent with the proliferation assay results, SKmel30 exhibited a reduced sensitivity to DH, although the reduction in viability remained significant (Supplementary Fig. 2B). Interestingly, in M161022 cells, DH and PP showed an even more pronounced inhibitory effect than Trametinib (Supplementary Fig. 3B). Drug effects were also evaluated in NRAS^mut^ melanoma cells embedded in a hydrogel matrix, to mimic the extracellular matrix (ECM) within the tumor microenvironment. Consistent with observations from scaffold-free spheroid and 3D aggregate cultures, DH and PP significantly inhibited the growth of SKmel147 (Supplementary Fig. 4A) and of the patient-derived cell line M161022 (Supplementary Fig. 4B).

Collectively, these findings demonstrate that DH and PP effectively suppress proliferation and reduce cell viability in a panel of NRAS^mut^ melanoma cell lines cultured under various 3D conditions.

### Pyrvinium Pamoate has a strong cytotoxic effect on NRAS^mut^ melanoma spheroids

Next, we evaluated whether the arrest of spheroid growth in response to DH or PP exposure was caused by cytotoxic effects of these compounds. After 5 days of treatment, spheroids and 3D aggregates were stained to assess the levels of activated executioner caspases 3 and 7 (CellEvent Caspase 3/7) and dead cells (SytoxBlue). Additionally, the expression of mCherry fluorescent protein in the transduced cells was used to identify viable tumor mass.

The level of apoptosis and cell death was linked to the tumor mass of the spheroid or cell aggregate on day 5 of treatment. Intrinsic apoptosis and cell death were observed in the inner core of untreated (UT) spheroids, aligning with the innate in vivo of 3D tumor formations [28]. Trametinib and DH exhibited low levels of cytotoxicity in SKmel147 (Fig. 3C) and SKmel30 cells (Supplementary Fig. 2C), suggesting a predominantly cytostatic effect. In contrast, PP induced apoptosis and cell death significantly in both cell lines, also indicated by a markedly reduced mCherry signal. Notably, in SKmel30, this reduction was even more pronounced than with STAU treatment (Supplementary Fig. 2C). Patient-derived metastatic melanoma cell lines exhibited similar cytotoxic responses across all treatments; with PP resulting in the greatest tumor mass reduction in spheroids and aggregates (Fig. 3C, Supplementary Fig. 3C & Fig. 5A).

In conclusion, our findings demonstrate that PP exerts a potent cytotoxic effect in a panel of established and patient-derived metastatic treatment-naïve NRAS^mut^ melanoma cell lines cultured under 3D conditions.

We evaluated the effects of the drugs on key proliferation and survival pathways in NRAS^mut^ melanoma, specifically ERK (MAPK pathway) and AKT (AKT pathway). ERK levels remained unchanged up to 5 days in SKmel147 (Fig. 3D-E) and SKmel30 cells (Supplementary Fig. 2D-E) across all treatments, indicating that DH and PP do not interfere with ERK expression. Similar findings were observed in the patient-derived melanoma cell lines M161022 (Supplementary Fig. 3D-E) and M160915 (Supplementary Fig. 5B-C). However, 5 days of PP treatment significantly affected the survival of patient-derived melanoma cells, resulting in insufficient lysate collection, underscoring the strong cytotoxic effect of PP in these cells.

As expected, phosphorylated ERK (pERK) was reduced by Trametinib in all cell lines (Fig. 3F, Supplementary Fig. 2F & 3F & 5D), consistent with its high specificity for MEK inhibition. However, treatment with DH and PP did not consistently alter pERK levels. For example, PP inhibited pERK in a time-dependent manner in SKmel147 (Fig. 3F) and M161022 (Supplementary Fig. 3F), but not in SKmel30 (Supplementary Fig. 2F) or M160915 (Supplementary Fig. 5D).

Neither Trametinib nor DH altered basal AKT expression across all cell lines, and phosphorylated AKT (pAKT, Ser473) exhibited inconsistent regulation in response to the drugs (Fig. 3G, Supplementary Fig. 2G & 3G & Fig. 5E). Notably, PP treatment consistently reduced total AKT levels in SKmel147 (Fig. 3D-E), M161022 (Supplementary Fig. 3D-E), and M160915 (Supplementary Fig. 5B-C). Due to this strong reduction in AKT expression, pAKT quantification was not feasible. In contrast, SKmel30 displayed a different response, with an increase in pAKT levels following PP treatment (Supplementary Fig. 2D-E and G). Given the pronounced reduction in AKT levels observed in SKmel147 and the patient-derived melanoma cell lines M161022 and M160915 upon PP treatment, we proceeded to evaluate the effect of PP on key downstream targets of AKT following a 3-day treatment period. First, we observed that PRAS40, a direct substrate of AKT and a negative regulator of mTORC1, was markedly reduced in PP-treated cells compared to untreated controls (Supplementary Fig. 4C). The reduction in total PRAS40 suggests a consequent loss of inhibition of mTORC1 activity. To further investigate the impact of PP on protein translation, we assessed key downstream effectors of mTORC1, including p70S6K and its direct substrate S6, as well as 4EBP1, a negative regulator of eIF4E. Across all three cell lines, PP treatment led to a reduction in p70S6K levels, accompanied by a marked decrease in S6 phosphorylation (Supplementary Fig. 4C). SKmel147 did not exhibit detectable S6 phosphorylation even under untreated conditions. Furthermore, PP treatment resulted in a pronounced reduction in 4EBP1 phosphorylation, with only a minimal decrease in total 4EBP1 protein levels (Supplementary Fig. 4C), suggesting enhanced inhibitory activity of 4EBP1 on eIF4E-mediated translation. In conclusion, our data suggest that PP treatment may impair protein translation in NRAS^mut^ melanoma cells.

Previous studies have reported that PP inhibits cancer cell growth by promoting β-Catenin degradation through its action as a CK1α agonist [24], whereas DH has been shown to induce DNA damage by intercalating into the DNA and causing double-strand breaks [25]. In line with this, we evaluated whether treatment with PP for 3 days (the earliest treatment time-point assessed) led to a reduction in nuclear β-Catenin levels in our melanoma cell lines. A decrease in nuclear β-Catenin was observed in SKmel147 (Fig. 3H) and SKmel30 (Supplementary Fig. 2H), whereas no significant reduction was detected in the two patient-derived cell lines (Supplementary Fig. 3H & 5 F). These results indicate that the effect of PP on β-Catenin downregulation is cell line–dependent. Conversely, DH effectively induced DNA damage, as evidenced by increased nuclear γH2AX expression in treated cells relative to untreated controls across all four melanoma cell lines (Fig. 3I, Supplementary Fig. 2I & 3I & 5G).

Overall, PP exerts an inhibitory effect on AKT protein levels, predominantly in patient-derived metastatic melanoma cell lines.

### Daunorubicin HCl and Pyrvinium Pamoate inhibit patient-derived melanoma cell invasion

The inhibitory effects of DH, PP, and Trametinib on the invasive abilities of melanoma cells were evaluated in SKmel147 and M160916 spheroids embedded in a type I Collagen matrix after 3 days of drug treatment. SKmel30 and M161022 were excluded from this assay due to their non-invasive phenotype and failure to form compact spheroids, respectively. In SKmel147, significant invasion inhibition occurred only with Trametinib, whereas DH and PP led to only minor reductions in cell motility (Supplementary Fig. 6A). In contrast, in the patient-derived melanoma cell line M160916, all three compounds significantly suppressed invasive activity (Supplementary Fig. 6B).

Although PP and DH did not reduce invasion in the established SKmel147 cell line, both compounds effectively inhibited the invasion of the patient-derived metastatic melanoma cell line.

### Combinatorial treatment with Trametinib (MEKi) shows additive effects

After assessing the effect of DH and PP as monotherapy on a panel of NRAS^mut^ melanoma cell lines, we explored their potential in combined treatments with Trametinib. This strategy was prompted by the well-studied ability of melanoma to develop resistance to monotherapies [29, 30]. We performed a 3D synergy assay on spheroids of 2 NRAS^mut^ melanoma cell lines, SKmel147 and SKmel30, to investigate potential synergistic effects between Trametinib and DH, and Trametinib and PP (Supplementary Fig. 7A). The range of concentrations of compounds (1:2 dilution ratio) was selected based on cell line-specific IC50 concentrations previously generated. Zero interaction potency (ZIP) synergy score analysis did not reveal overall synergism (defined as ZIP synergy score > 10); however, an additive effect (defined as ZIP synergy score >-10 and < 10) was consistently observed across all conditions. For the following experiments, we selected drug concentrations determining the regions of maximum synergistic effects: Trametinib at 0.06 nM and DH and PP at 45 nM.

We subsequently assessed the impact of these combinations on SKmel147 and SKmel30 spheroid proliferation and cell viability in parallel with single synergy concentration treatments. Despite using drug concentrations belonging to the region with the highest synergism, the results remained additive, showing only a slight reduction in proliferation (Supplementary Fig. 7B-D) and cell viability (Supplementary Fig. 7C-E) compared to the respective single treatments in both cell lines. The drug combinations exhibited good effects on SKmel147 cell proliferation (Supplementary Fig. 7B) and spheroid viability (Supplementary Fig. 7C) but did not affect SKmel30 growth (Supplementary Fig. 7D-E), consistent with previous observations on the intrinsic resistance of this cell line. Overall, the combinatorial treatment at theoretically synergistic concentrations yielded less effective and more inconsistent outcomes compared to monotherapy at IC50 concentrations.

Based on these results, we further concentrated on the characterization of the monotherapies (based on the cell line-specific IC50 values) using advanced *in vitro* pre-clinical models.

### Advanced in vitro 3D co-culture models reveal melanoma-specific effects and low toxicity of Daunorubicin HCl and Pyrvinium Pamoate

**Fig. 4:**
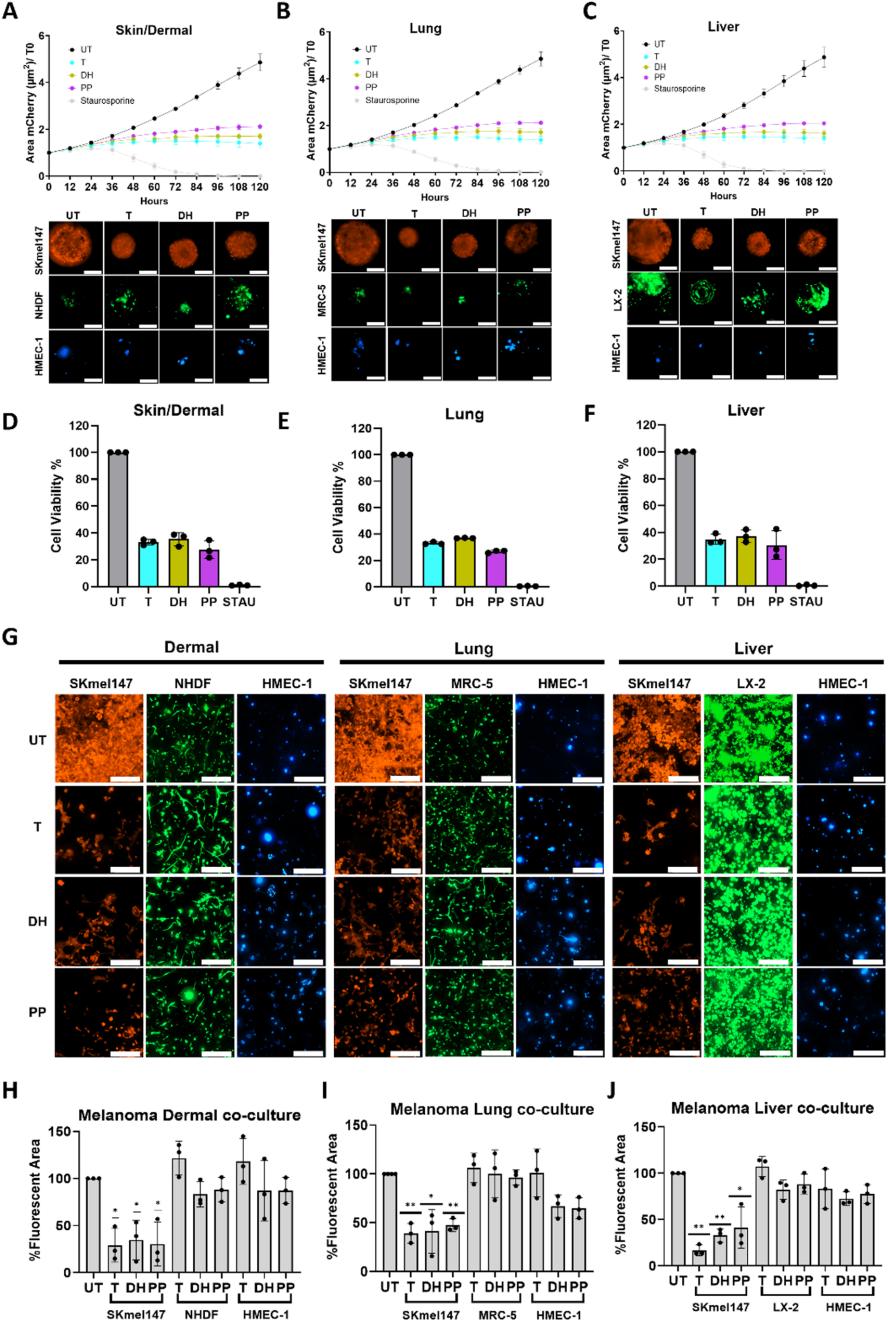
SKmel147-TME co-culture models show inhibitory melanoma-specific effect and low toxicity on non-cancerous cells. **A-C**) Upper panels: Kinetic response of SKmel147-mCherry co-cultures to 5-day drug treatment in 3 different Melanoma Multicomponent Spheroid (MMS) models: “Skin/Dermal” (**A**), “Lung” (**B**), and Liver” (**C**). Images of mCherry fluorescence were acquired every 12 h. The spheroid area was determined and plotted. Lower panels: corresponding confocal images (20x magnification) of the different cell populations after 5 days of drug treatment. Scale bar = 200 μm. (*n* = 3. mean ± SD) **D-F**) Cell viability of the 3 SKmel147-MMS models after 5 days of drug treatment. Data are normalized to untreated control. Staurosporine was used as positive control at 200 nM in A-F. (*n* = 3. mean ± SD). **G**) Representative confocal pictures (20x magnification) of 3 hydrogel-embedded SKmel147-TME co-culture models (“Dermal”, “Lung”, and Liver”) after 5-day treatment: SKmel147-mCherry (red), NHDF/MRC-5/LX-2 (green), HMEC-1 (blue). Scale bar = 200 μm. **H-J**) Plots representing the percentage of fluorescent area of the different cell populations in the 3 hydrogel co-culture models. Data are normalized to the untreated control of each specific cell population. One sample T-test was used for **H-J** for statistical significance testing (*n* = 3. mean ± SD; *p ≤ 0.05, **p ≤ 0.01). Melanoma cell line-specific IC50 concentrations of Daunorubicin HCl (DH), Pyrvinium Pamoate (PP) and Trametinib (T) were used.

The role of non-cancerous cells in the tumor microenvironment (TME) in supporting cancer survival and drug resistance is well established [10, 31, 32]. Co-culture models are valuable for assessing drug efficacy by capturing cancer cell–TME interactions and evaluating toxicity on non-cancerous cells. Using our previously established Multicomponent Melanoma Spheroid (MMS) models [11], which mimic key metastatic sites such as “skin/dermal” (HMEC-1 + NHDF), “lung” (HMEC-1 + MRC-5), and “liver” (HMEC-1 + LX-2), we assessed the effects of DH and PP. The cellular ratio of 1:3:3 (melanoma: fibroblasts: endothelial cells) and the medium used for the co-culture were established based on optimization experiments recently published by our group [11]. Fluorescent labeling allowed real-time visualization of cell populations by extracting the residual MIP spheroid area. Time-lapse microscopy showed reduced SKmel147 proliferation in all MMS models compared to untreated controls (Fig. 4A-C). To a similar extent, DH, PP, and Trametinib reduced total co-culture viability (Fig. 4D-F), with 30–40% residual viability attributed to melanoma while TME normal cells survived (Fig. 4A-C). SKmel30 showed similar proliferation inhibition in the “dermal” (Supplementary Fig. 8A) and “lung” (Supplementary Fig. 8B) models but less in the “liver” models (Supplementary Fig. 8C) compared to monocomponent spheroids (Supplementary Fig. 2A). In line with the monocomponent data, DH had a weaker inhibitory effect on SKmel30 proliferation than Trametinib and PP. Corresponding viability assays showed significant reductions, with DH presenting the lowest efficacy compared to Trametinib and PP (Supplementary Fig. 8D-F), with residual viability due to melanoma and TME cells (Supplementary Fig. 8A-C).

We next investigated the effects of DH and PP in complex models incorporating an extracellular matrix (ECM), a critical factor in melanoma progression and drug resistance [33, 34]. Using hydrogel-embedded melanoma-TME co-culture models, we evaluated the efficacy of these drugs alongside Trametinib while also assessing their effects on non-cancerous cells. Fluorescent labeling enabled clear visualization of melanoma and TME cell populations. In these models, SKmel147 showed significant growth reductions across all conditions (Fig. 4G-J). In the “dermal” (Fig. 4H) and “lung” (Fig. 4I) models, all three drugs reduced the melanoma population by over 50% compared to controls. The “liver” (Fig. 4J) model revealed increased sensitivity of SKmel147 to Trametinib, compared to the other models, and to the monoculture (Supplementary Fig. 4A). Interestingly, DH and especially PP significantly inhibited M161022 growth across all three models (Supplementary Fig. 9A-D), in line with what was observed in monoculture (Supplementary Fig. 4B). The survival of non-cancerous TME cells was also evaluated. Importantly, drug concentrations effective against melanoma cells had a low impact on the survival of non-cancerous cells in co-culture, highlighting the potent melanoma inhibitory effects and safety profile of these compounds.

### Trametinib inhibitor-resistant melanoma cells are sensitive to Daunorubicin HCl and pyrvinium pamoate

Given melanoma’s rapid development of resistance to current therapies, we have generated NRAS^mut^ (SKmel30) and WT (WM3918) melanoma cell lines resistant to Trametinib (Tres) and evaluated the efficacy of DH and PP in resistant cells. Of note, SKmel147 has not developed resistance, even after prolonged drug exposure (approximately 6 months), illustrating the high heterogeneity between melanoma cells and has therefore not been included in the following experiments.

**Fig. 5:**
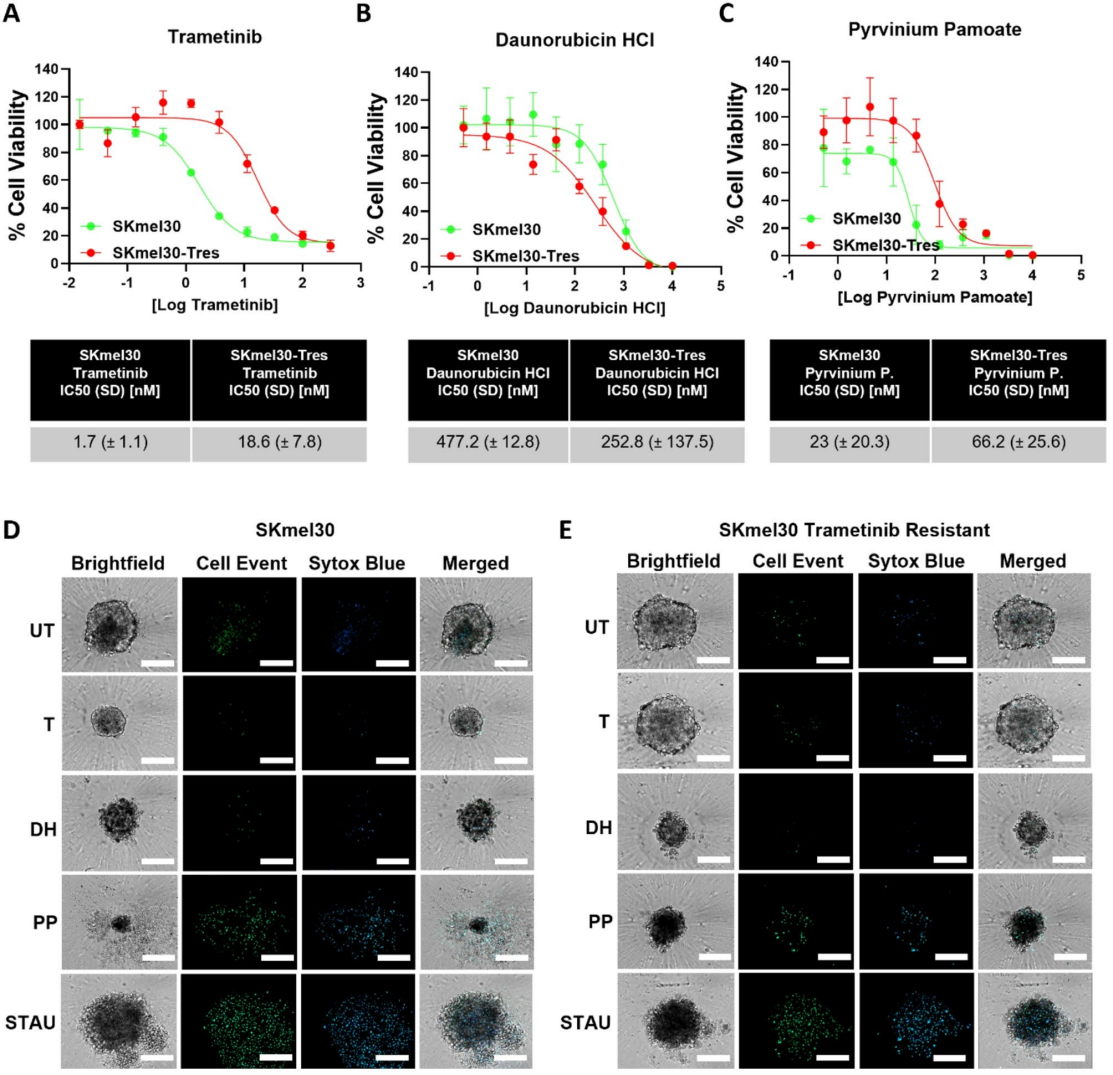
Daunorubicin HCl and Pyrvinium Pamoate exert inhibitory and cytotoxic effect on Trametinib-resistant NRAS^mut^ melanoma cell lines. **A-C**) Representative drug response curves for Trametinib (MEKi) (**A**), Daunorubicin HCl (**B**) and Pyrvinium Pamoate (**C**) in Trametinib-sensitive (green) and -resistant (red) SKmel30 cells cultured as spheroids, utilizing CellTiter-Glo^®^ 3D Cell Viability Assay as readout after 5 days of treatment. Reported IC50 values in the tables are mean ± SD of 3 independent biological replicates. Tres: Trametinib-resistant. **D-E**) Representative photos of apoptosis and cell death detection in SKmel30 (**D**) and SKmel30 T-res (**E**) spheroids after 5 days of treatment. Staurosporine was used as positive control at 1µM. Apoptosis (green) and cell death (blue) were measured upon the addition of the CellEvent Caspase-3/7 and Sytox Blue detection reagents, respectively. Confocal images (20x magnification) of single spheroids are shown. Scale bar = 200 μm (*n* = 3)

DRCs of sensitive and resistant cell lines cultured as spheroids and relative IC50 values were generated for DH, PP, and Trametinib after 5 days of treatment. Resistance to Trametinib was confirmed by increased IC50 values in both SKmel30-Tres (Fig. 5A) and WM3918-Tres cells (Supplementary Fig. 10A) in comparison to the sensitive counterparts.

A 3D apoptosis/cell death assay further showed the low cytotoxic effect of Trametinib in sensitive SKmel30 cells (Fig. 5D), consistent with previous findings (Supplementary Fig. 2C), as well as in SKmel30-Tres cells (Fig. 5E). A similar effect was observed in WM3918-Tres cells (Supplementary Fig. 10E), whereas Trametinib treatment exhibited cytotoxicity on sensitive WM3918 cells (Supplementary Fig. 10D). Although DH appeared to be more effective in SKmel30-Tres cells compared to their sensitive counterparts, as indicated by a lower IC50 value (Fig. 5B), it did not induce substantial levels of apoptosis or cell death after 5 days of treatment (Fig. 5E).

In contrast, WM3918 cells displayed a significant increase in sensitivity to DH, as demonstrated by a reduction in IC50 values in WM3918-Tres cells (116.6 nM) compared to their sensitive counterparts (743.6 nM) (Supplementary Fig. 10B). This was further supported by the strong cytotoxic effect of DH observed in both WM3918 sensitive (Supplementary Fig. 10D) and WM3918-Tres cells (Supplementary Fig. 10E). Consistent with previous findings, PP induced high levels of apoptosis and cell death following 5 days of treatment in both sensitive SKmel30 cells (Fig. 5D) and SKmel30-Tres cells (Fig. 5E) compared to Trametinib and DH. Images revealed a reduced spheroid size and lower cytotoxic activity of PP in SKmel30-Tres cells compared to SKmel30 sensitive cells, which was further corroborated by IC50 values, where SKmel30-Tres cells exhibited a higher IC50 value (66.2 nM) than SKmel30 sensitive cells (23 nM) (Fig. 5C).

Despite comparable growth inhibition of WM3918 sensitive and WM3918-Tres cells by PP (Supplementary Fig. 10C), PP exhibited a pronounced cytotoxic effect in WM3918-Tres cells (Supplementary Fig. 10E), which was not observed in their sensitive counterparts (Supplementary Fig. 10D).

These findings suggest that DH and PP hold potential as second-line therapeutic agents for targeting melanoma cells that have developed resistance to targeted therapies such as MEKi.

### Daunorubicin HCl and Pyrvinium Pamoate show strong inhibitory effects in zebrafish xenograft melanoma models

**Fig. 6:**
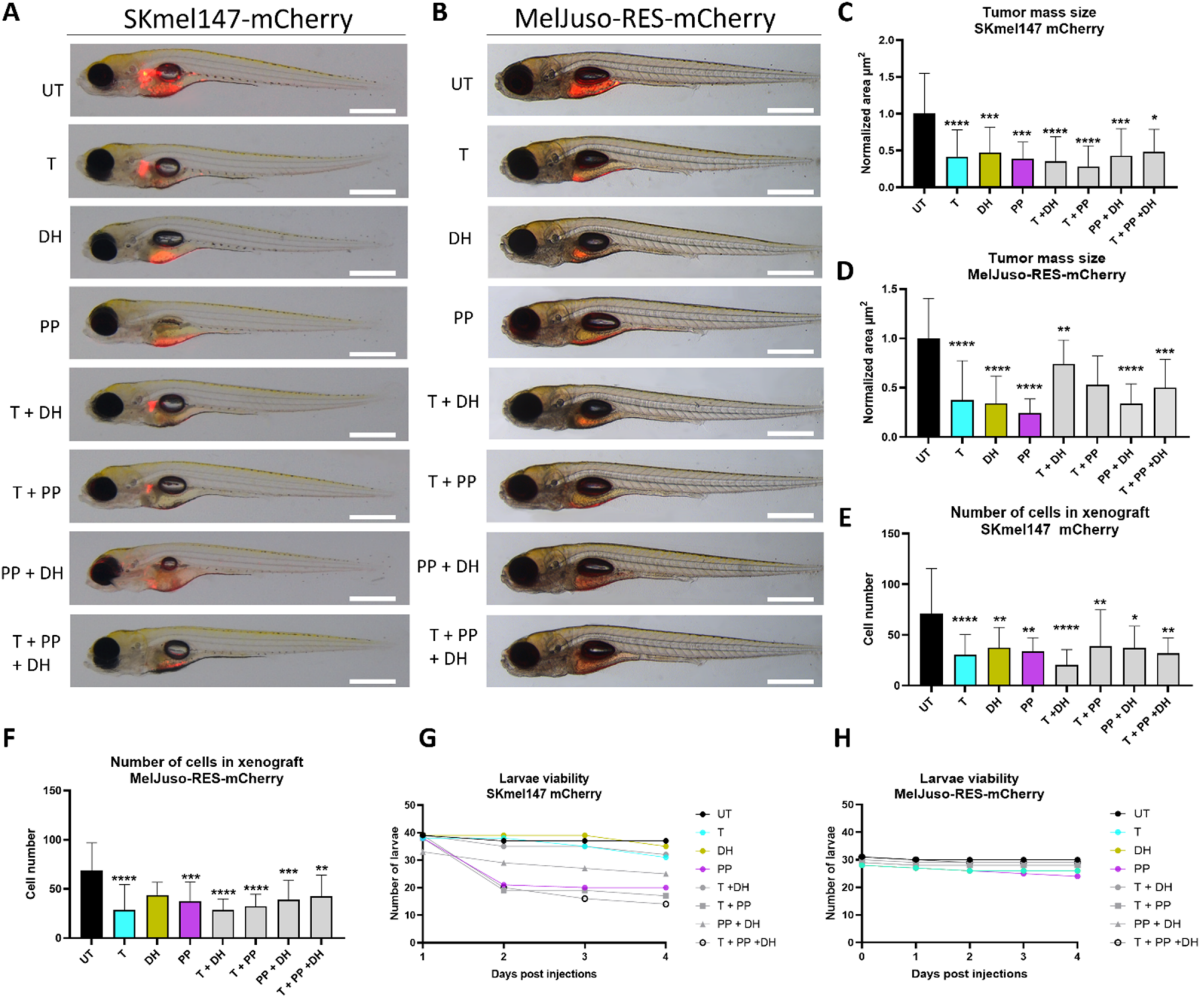
Daunorubicin HCl and Pyrvinium Pamoate show strong melanoma inhibitory effects in zebrafish xenograft models. Left panel: SKmel147-mCherry xenografts. Right panel: MelJuso-RES-mCherry xenografts. Skmel147-mCherry (**A**) and MelJuso-RES-mCherry cells (**B**) were injected into the yolk of 2dpf zebrafish and subjected to mono- or combinatory treatments. Scale bar: 500 μm. The xenograft area (**C-D**) and the number of cells per xenograft (**E-F**) were evaluated after 3 days of treatment based on the mCherry signal by two independent investigators. Graphs represent the mean ± SD of normalized data. Statistical significance was assessed with Shapiro-Wilk normality test followed by Kruskal Wallis test with Dunn’s multiple comparisons: ∗*p* < 0.05; ∗∗*p* < 0.01; ∗∗∗*p* < 0.001; ∗∗∗∗*p* < 0.00001. (**G-H**) Larvae viability was monitored daily over the course of the treatment. MelJuso-RES-mCherry cells: Binimetinib-resistant NRAS^mut^ melanoma cells.

To assess the in vivo efficacy of the drugs, SKmel147-mCherry and MelJuso-RES-mCherry cell lines were injected into the yolks of 2-day post-fertilization *nacre* zebrafish larvae. As SKmel30-Tres cells used in *in vitro* experiments failed to form proper tumors post-injection, MelJuso-RES-mCherry cells have been chosen to represent the resistant melanoma phenotype. At 24 h post-injection, larvae were randomized into groups of 30–40 individuals and treated with the drugs as monotherapy or in combination treatments at doses previously established as the maximum tolerated dose. After 72 h of treatment, larval survival, metastasis status and xenograft size were evaluated. Despite the highly invasive and motile phenotype of the melanoma cells, no increase in migration from the initial injection site was detected (Fig. 6A-B). Consistent with *in vitro* findings, a significant reduction in xenograft area was observed in both sensitive and resistant cell lines. Notably, this effect was evident not only in monotherapy groups (Trametinib, DH, and PP) but also in combination treatments (Fig. 6C-D). The number of injected cells in the untreated control remained stable over the 72-hour period, whereas a significant reduction was observed in the treated groups, indicating strong cytotoxic effects of the tested drugs in vivo (Fig. 6E-F). While no increased mortality was noted in MelJuso-RES-mCherry injected larvae, some mortality was observed in SKmel147 injected larvae, particularly in groups treated with PP and its combinations, despite the doses being within the previously established safe range for larval survival and development (Fig. 6G-H).

## Discussion

Systemic therapy for melanoma has advanced significantly, with targeted and immune therapies primarily benefiting BRAF^mut^ patients [35]. However, NRAS^mut^ melanoma patients only rely on ICIs as a first-line treatment, with response rates below 50% [4, 7], and off-label MEKi, like Trametinib, as a second-line treatment if ICI must be discontinued. Hence, additional novel therapeutic options for NRAS^mut^ patients are urgently needed.

To address this, we conducted high-throughput screening (HTS) to evaluate more than 1300 compounds. Unlike traditional HTS on adherent cell cultures, we used 3D melanoma spheroids to improve physiological relevance and mimic patient tumor responses more accurately [36].

Hit identification followed a rigorous multi-step process, which led to the identification of 17 promising compounds with strong inhibitory effects on NRAS^mut^ melanoma cells in 3D spheroids, warranting their further investigation as potential melanoma therapies. A limitation of this study is the use of only one cell line for screening. However, given the increased complexity of the 3D culture model, using a single cell line served as a practical and reasonable foundation for identifying potential candidate compounds.

Among the identified hits, DH and PP emerged as the most promising candidates. DH, an anthracycline antibiotic [37], is approved for acute myeloid leukemia (AML) [25, 38]. It acts as a topoisomerase II (TOP2) poison, inducing DNA damage and apoptosis [25, 39, 40]. In melanoma, Mu et al. demonstrated that TOP2α is significantly overexpressed compared to benign nevi [41]. PP is a cyanine dye approved as an anthelmintic drug [24]. Its mechanism of action is not yet fully elucidated, however some studies on cancer describe PP as a CK1α agonist that promotes β-Catenin degradation [24, 42], a mechanism also observed in uveal melanoma [43].

DH and PP were tested in dose-response assays in NRAS^mut^ melanoma cell lines, showing high efficacy in 3D spheroid cultures. Both demonstrated a favorable safety profile in non-cancerous cells, particularly PP, consistent with their FDA approval status. Despite significant viability reduction, their effects appeared largely cytostatic, as proliferation decreased but remained above baseline. All melanoma cell lines responded similarly, except SKmel30, which exhibited greater resistance to DH, likely due to a *TP53* deletion (Cellosaurus.org). This aligns with findings that mutant p53 reduces the efficacy of TOP1 and TOP2 inhibitors [44]. Furthermore, Dunsche et al. demonstrated that a rare *TP53* mutation (R285K) confers increased resistance to cisplatin treatment in metastatic melanoma cells. This resistance can be overcome through ferroptosis induction [45]. On the other hand, we showed that PP induced apoptosis and cell death across all cell lines. In contrast, Trametinib and DH triggered lower cytotoxic effects, particularly in SKmel30, which exhibited general resistance, as observed in the Staurosporine-treated spheroids. Apoptosis and cell death were evaluated after five days of treatment; therefore, DH and Trametinib may have induced cytotoxicity at an earlier time point or activated alternative programmed cell death pathways, such as necroptosis [46, 47]. However, translational cancer therapies primarily depend on prolonged treatment regimens rather than short-term interventions.

Mechanistically, we focused on the deregulation of key pathways involved in NRAS^mut^ melanoma survival, which are often rewired in response to targeted therapy treatments, leading to drug resistance. These pathways include the MAPK and AKT pathways [48–50]. As expected, Trametinib induced a general reduction in ERK activation due to its inhibitory action on MEK, the upstream kinase of ERK. In contrast, DH exhibited inconsistent effects on the deregulation of pERK and pAKT across the cell lines, suggesting the involvement of other major targets. Notably, PP reduced AKT levels in SKmel147 and patient-derived cell lines, positioning it as a potential AKT regulator. PP’s role in AKT inhibition in cancer has been reported [51–53], also in uveal melanoma [43]. Given PP’s apoptotic effects, AKT may contribute to this by downregulating anti-apoptotic molecules like BCL-2 and BCL-xL and affecting mitochondrial stability [50, 54]. We also observed a notable inhibitory effect of PP in NRAS^mut^ melanoma cell lines, characterized by the downregulation of key components of the AKT–mTORC1 pathway involved in protein translation, including p70S6K, S6, and 4EBP1 [55].

Additionally, investigation of the effects of PP and DH on β-Catenin and DNA damage, respectively, revealed that PP inhibited nuclear β-Catenin in two out of four melanoma cell lines, indicating a cell line–dependent effect. In contrast, all DH-treated melanoma cell lines exhibited a significant increase in γH2AX expression. Overall, additional studies are needed to fully understand DH and PP’s effects on melanoma.

We evaluated DH and PP for their ability to inhibit cell invasion using spheroids embedded in a type I Collagen matrix. Trametinib suppressed invasion in both melanoma cell lines, contrasting a study by Vultur et al., who found that MEKi increased motility in metastatic but not non-metastatic melanoma [56]. Notably, DH and PP significantly reduced invasive behavior only in the patient-derived metastatic cell line (M160915), suggesting their effects may be phenotype-specific in NRAS^mut^ melanoma.

To evaluate compound effects within the tumor microenvironment, DH and PP were tested in co-culture systems, including Melanoma Multicomponent Spheroids (MMS) [11] and a hydrogel-based melanoma-TME system. NRAS^mut^ melanoma cell lines were co-cultured with endothelial cells and fibroblasts (NHDF for skin/dermal, MRC-5 for lung, LX-2 for liver) and treated with Trametinib, DH, and PP at IC50 concentrations. While SKmel147 and SKmel30 responded similarly to treatments in skin/dermal and lung models, SKmel30 exhibited increased resistance to PP in the liver model. A more advanced 3D hydrogel system revealed that DH and PP selectively inhibited melanoma cell growth with minimal impact on non-cancerous cells. This effect was stronger than with Trametinib in the M161022 model. Notably, SKmel147 responded differently in the liver model, highlighting treatment response variations based on metastatic sites. This aligns with Forschner et al.‘s findings that response to targeted therapies and immune checkpoint inhibitors varies by metastatic site [57].

It is well known that melanoma develops resistance to treatments in the majority of cases, leading to relapses and leaving patients, especially NRAS^mut^, without efficient treatment options. To overcome this, we evaluated DH and PP in Trametinib (MEKi)-resistant NRAS^mut^ and BRAF^wt^/NRAS^wt^ melanoma cell lines. Importantly, PP effectively inhibited Trametinib-resistant melanoma growth, while DH showed an even greater effect than in non-resistant cells. Although preliminary, our results support the potential of both compounds as a second- or third-line treatment for MEKi-resistant patients, and we are currently following this up by generating more melanoma cell lines resistant to targeted therapies and eventually to ICIs.

Final validation of DH and PP anti-melanoma efficacy was performed in a zebrafish xenograft model. Recently, such models have been established and proved to be valid for melanoma development, drug screening and resistance mechanism in vivo studies. It provides a relevant physiological background and offers ethical advantages over mice adhering to the 3R principles (Replacement, Reduction, Refinement) [58–60]. This validation confirmed DH and PP anti-tumor effects in sensitive and MEKi-resistant NRAS^mut^ melanoma models with low toxicity, enhancing their translational effect.

Overall, given its role in AKT inhibition, apoptosis, and cell death induction, PP stands as a promising first-line therapy for NRAS^mut^ melanoma, warranting further clinical investigation.

One key challenge is PP’s current tablet formulation, which limits systemic absorption. However, Esumi et al. reported intestinal absorption of PP, leading to reduced pancreatic tumor size in mice [61]. Furthermore, we hypothesize that DH could be effectively utilized in an immunotherapy-rechallenging setting, given its potential as a chemotherapeutic anthracycline compound capable of inducing immunogenic cell death [46], thereby enhancing the anti-cancer immune response [62]. This hypothesis is further supported by the findings of Gebhardt et al., who demonstrated that low doses of paclitaxel increased the presence of functional cytotoxic T-cells while reducing tumor-suppressive MDSCs (myeloid-derived suppressor cells), ultimately resensitizing patients resistant to immune checkpoint inhibitors [63].

## Conclusions

In this study, we performed high-throughput drug screening using 3D NRAS^mut^ melanoma spheroid cultures, improving the translational relevance of drug responses over traditional 2D models. Among the over 1300 compounds tested, Daunorubicin HCl and Pyrvinium Pamoate emerged as effective against NRAS^mut^ melanoma, demonstrating growth inhibition in advanced 3D *in vitro* models and zebrafish xenografts, paving the way for their potential consideration either alone or in combination with other therapies. Finally, we also introduced that the strategic combination of repurposed, clinically approved drugs with advanced human 3D models holds considerable potential to both accelerate the drug discovery process and improve drug approval success rates.

## Supplementary Information

Below is the link to the electronic supplementary material.

Supplementary Material 1

## Data Availability

No datasets were generated or analysed during the current study.

## Abbreviations

NRAS: Neuroblastoma RAS viral oncogene homolog
BRAF: V-Raf Murine Sarcoma Viral Oncogene Homolog B
WT: Wild Type
TME: Tumor Microenvironment
ECM: Extracellular Matrix
HTS: High-Throughput Screening
MIP: Maximum Intensity Projection
ULA: Ultra Low Attachment
ZIP: Zero Interaction Potency
DRC: Drug-Response Curve
IC50: Half-maximal inhibitory concentration
MMS: Melanoma Multicomponent Spheroid
DH: Daunorubicin HCl
PP: Pyrvinium Pamoate
MEK: Mitogen-activated protein kinase kinase
ERK: Extracellular signal-regulated kinase
AKT: Protein Kinase B or PKB or Ak strain transforming

## References

[1] Long GV, Swetter SM, Menzies AM, Gershenwald JE, Scolyer RA. Cutaneous melanoma. Lancet. 2023;402(10400):485–502. 37499671 10.1016/S0140-6736(23)00821-8

[2] Shain AH, Bastian BC. From melanocytes to melanomas. Nat Reviews Cancer Nat Publishing Group. 2016;16:345–58. 10.1038/nrc.2016.37 27125352

[3] Akbani R, Akdemir KC, Aksoy BA, Albert M, Ally A, Amin SB et al. Genomic Classification of Cutaneous Melanoma. Cell. 2015;161(7):1681–96. Available from: https://linkinghub.elsevier.com/retrieve/pii/S0092867415006340 10.1016/j.cell.2015.05.044 PMC4580370 26091043

[4] Boutros A, Croce E, Ferrari M, Gili R, Massaro G, Marconcini R et al. The treatment of advanced melanoma: Current approaches and new challenges. Crit Rev Oncol Hematol. 2024;196:104276. Available from: https://linkinghub.elsevier.com/retrieve/pii/S1040842824000192 10.1016/j.critrevonc.2024.104276 38295889

[5] Fernandez MF, Choi J, Sosman J. New approaches to targeted therapy in melanoma. Cancers (Basel). 2023;15(12):3224. 37370834 10.3390/cancers15123224 PMC10296143

[6] Randic T, Kozar I, Margue C, Utikal J, Kreis S. NRAS mutant melanoma: Towards better therapies. Cancer Treat Rev. 2021/06/08. 2021;99:102238. Available from: https://www.ncbi.nlm.nih.gov/pubmed/34098219 10.1016/j.ctrv.2021.102238 34098219

[7] Amaral T, Ottaviano M, Arance A, Blank C, Chiarion-Sileni V, Donia M et al. Cutaneous melanoma: ESMO clinical practice guideline for diagnosis, treatment and follow-up. Ann Oncol. 2024. 10.1016/j.annonc.2024.11.006 PMC7618628 39550033

[8] Kirchberger MC, Ugurel S, Mangana J, Heppt MV, Eigentler TK, Berking C, et al. MEK Inhibition May increase survival of NRAS-mutated melanoma patients treated with checkpoint blockade: results of a retrospective multicentre analysis of 364 patients. Eur J Cancer. 2018;98:10–6. 29843107 10.1016/j.ejca.2018.04.010

[9] Edmondson R, Broglie JJ, Adcock AF, Yang L. Three-Dimensional cell culture systems and their applications in drug discovery and cell-Based biosensors. Assay Drug Dev Technol. 2014;12(4):207–18. 24831787 10.1089/adt.2014.573 PMC4026212

[10] Pape J, Emberton M, Cheema U. 3D cancer models: the need for a complex stroma, compartmentalization and stiffness. Frontiers in Bioengineering and Biotechnology. Frontiers Media S.A.; 2021, 9. 10.3389/fbioe.2021.660502 PMC8072339 33912551

[11] Angeli C, Wroblewska JP, Klein E, Margue C, Kreis S. Protocol to generate scaffold-free, multicomponent 3D melanoma spheroid models for preclinical drug testing. STAR Protoc. 2024;5(2):103058. Available from: https://linkinghub.elsevier.com/retrieve/pii/S2666166724002235 10.1016/j.xpro.2024.103058 PMC11109877 38748881

[12] Kulkarni VS, Alagarsamy V, Solomon VR, Jose PA, Murugesan S. Drug repurposing: an effective tool in modern drug discovery. Russ J Bioorg Chem. 2023;49(2):157–66. 36852389 10.1134/S1068162023020139 PMC9945820

[13] Hughes J, Rees S, Kalindjian S, Philpott K. Principles of early drug discovery. Br J Pharmacol. 2011;162(6):1239–49. 21091654 10.1111/j.1476-5381.2010.01127.x PMC3058157

[14] Malo N, Hanley JA, Cerquozzi S, Pelletier J, Nadon R. Statistical practice in high-throughput screening data analysis. Nat Biotechnol. 2006;24(2):167–75. 16465162 10.1038/nbt1186

[15] Margue C, Philippidou D, Kozar I, Cesi G, Felten P, Kulms D et al. Kinase inhibitor library screening identifies synergistic drug combinations effective in sensitive and resistant melanoma cells, Journal of Experimental and Clinical Cancer Research. Journal of Experimental & Clinical Cancer Research. 2019;38:1–17. Available from: https://jeccr.biomedcentral.com/track/pdf/10.1186/s13046-019-1038-x.pdf 10.1186/s13046-019-1038-x PMC6364417 30728057

[16] Cesi G, Walbrecq G, Zimmer A, Kreis S, Haan C. ROS production induced by BRAF inhibitor treatment rewires metabolic processes affecting cell growth of melanoma cells. Mol Cancer. 2017;16(1):102. 28595656 10.1186/s12943-017-0667-y PMC5465587

[17] Ehrbar M, Rizzi SC, Hlushchuk R, Djonov V, Zisch AH, Hubbell JA, et al. Enzymatic formation of modular cell-instructive fibrin analogs for tissue engineering. Biomaterials. 2007;28(26):3856–66. 17568666 10.1016/j.biomaterials.2007.03.027

[18] Westerfield M. The zebrafish book. A guide for the laboratory use of zebrafish (Danio rerio). 4th Edition. Univ. of Oregon Press Eugene, editor. 2000.

[19] Aleström P, D’Angelo L, Midtlyng PJ, Schorderet DF, Schulte-Merker S, Sohm F, et al. Zebrafish: housing and husbandry recommendations. Lab Anim. 2020;54(3):213–24. 31510859 10.1177/0023677219869037 PMC7301644

[20] Schneider NFZ, Cerella C, Simões CMO, Diederich M. Anticancer and Immunogenic properties of cardiac glycosides. Molecules: MDPI AG; 2017, 22. 10.3390/molecules22111932 PMC6150164 29117117

[21] Kumavath R, Paul S, Pavithran H, Paul MK, Ghosh P, Barh D, et al. Emergence of cardiac glycosides as potential drugs: current and future scope for cancer therapeutics. Biomolecules. MDPI AG; 2021, 11. 10.3390/biom11091275 PMC8465509 34572488

[22] Gajos-Michniewicz A, Czyz M. Wnt signaling in melanoma. International Journal of Molecular Sciences. MDPI AG. 2020;21:1–31. 10.3390/ijms21144852 PMC7402324 32659938

[23] Xue G, Romano E, Massi D, Mandalà M. Wnt/β-catenin signaling in melanoma: preclinical rationale and novel therapeutic insights. Cancer Treatment Reviews. W.B. Saunders Ltd; 2016;49:1–12. 10.1016/j.ctrv.2016.06.009 27395773

[24] Christopher W, Schultz, Avinoam Nevler. Pyrvinium Pamoate: Past, Present, and Future as an Anti-Cancer Drug. Biomedicines. 2022. 10.3390/biomedicines10123249 PMC9775650 36552005

[25] Mattioli R, Ilari A, Colotti B, Mosca L, Fazi F, Colotti G. Doxorubicin and other anthracyclines in cancers: activity, chemoresistance and its overcoming. Mol Aspects Med. 2023;93. 10.1016/j.mam.2023.101205 37515939

[26] Dsouza R, Jain M, Khattar E. p53-deficient cancer cells hyperactivate DNA double-strand break repair pathways to overcome chemotherapeutic damage and augment survival. Mol Biol Rep. 2025;52(1):333. 40119972 10.1007/s11033-025-10434-1

[27] Kumar RJ, Chao HX, Simpson DA, Feng W, Cho MG, Roberts VR et al. Dual Inhibition of DNA-PK and DNA polymerase theta overcomes radiation resistance induced by p53 deficiency. NAR Cancer. 2020;2(4). 10.1093/narcan/zcaa038 PMC7751686 33385162

[28] Cui X, Hartanto Y, Zhang H. Advances in multicellular spheroids formation. J R Soc Interface. 2017/02/17. 2017;14(127). Available from: https://www.ncbi.nlm.nih.gov/pubmed/28202590 10.1098/rsif.2016.0877 PMC5332573 28202590

[29] Czarnecka AM, Bartnik E, Fiedorowicz M, Rutkowski P. Targeted therapy in melanoma and mechanisms of resistance. International Journal of Molecular Sciences. MDPI AG; 2020;21:1–21. 10.3390/ijms21134576 PMC7369697 32605090

[30] Kozar I, Margue C, Rothengatter S, Haan C, Kreis S. Many ways to resistance: how melanoma cells evade targeted therapies. Biochimica et Biophysica Acta - Reviews on Cancer. Elsevier; 2019;1871:313–22. 10.1016/j.bbcan.2019.02.002 30776401

[31] Rodrigues J, Heinrich MA, Teixeira LM, Prakash J. 3D In Vitro Model (R)evolution: Unveiling Tumor-Stroma Interactions. Trends Cancer. 2020/11/22. 2021;7(3):249–64. Available from: https://www.ncbi.nlm.nih.gov/pubmed/33218948 10.1016/j.trecan.2020.10.009 33218948

[32] Nascentes Melo LM, Kumar S, Riess V, Szylo KJ, Eisenburger R, Schadendorf D et al. Advancements in melanoma cancer metastasis models. Pigment Cell and Melanoma Research. John Wiley and Sons Inc; 2023;36:206–23. 10.1111/pcmr.13078 36478190

[33] Popovic A, Tartare-Deckert S. Role of extracellular matrix architecture and signaling in melanoma therapeutic resistance. Front Oncol. 2022;12. 10.3389/fonc.2022.924553 PMC9479148 36119516

[34] Cox TR. The matrix in cancer. Nat Reviews Cancer Nat Res. 2021;21:217–38. 10.1038/s41568-020-00329-7 33589810

[35] Augustin RC, Luke JJ. Top advances of the year: Melanoma. Cancer. John Wiley and Sons Inc; 2023;129:822–8. 10.1002/cncr.34590 PMC11234509 36629350

[36] Barbosa MAG, Xavier CPR, Pereira RF, Petrikaitė V, Vasconcelos MH. 3D cell culture models as recapitulators of the tumor microenvironment for the screening of Anti-Cancer drugs. Cancers. MDPI; 2022, 14. 10.3390/cancers14010190 PMC8749977 35008353

[37] Aubel-Sadron G, Londos-Gagliardi D. Daunorubicin and doxorubicin, anthracycline antibiotics, a physicochemical and biological review. BIOCHIMIE. 1984, 66. 10.1016/0300-9084(84)90018-x 6380596

[38] Wang H, Xiao X, Xiao Q, Lu Y, Wu Y. The efficacy and safety of Daunorubicin versus Idarubicin combined with cytarabine for induction therapy in acute myeloid leukemia: A meta-analysis of randomized clinical trials. Lippincott Williams and Wilkins; 2020;99:E20094. Medicine (United States). 10.1097/MD.0000000000020094 PMC7302600 32541448

[39] Buzun K, Bielawska A, Bielawski K, Gornowicz A. DNA topoisomerases as molecular targets for anticancer drugs. Journal of Enzyme Inhibition and Medicinal Chemistry. Taylor and Francis Ltd. 2020;35:1781–99. 10.1080/14756366.2020.1821676 PMC7534307 32975138

[40] Al-Aamri HM, Ku H, Irving HR, Tucci J, Meehan-Andrews T, Bradley C. Time dependent response of Daunorubicin on cytotoxicity, cell cycle and DNA repair in acute lymphoblastic leukaemia. BMC Cancer. 2019;19(1). 10.1186/s12885-019-5377-y PMC6391779 30813936

[41] Mu XC, Tran TA, Ross JS, Carlson JA. Topoisomerase II-alpha expression in melanocytic nevi and malignant melanoma. J Cutan Pathol. 2000;27(5):242–8. 10847549 10.1034/j.1600-0560.2000.027005242.x

[42] Fan J, Reid RR, He TC. Pyrvinium doubles against WNT-driven cancer. Journal of Biological Chemistry. American Society for Biochemistry and Molecular Biology Inc. 2022, 298. 10.1016/j.jbc.2022.102479 PMC9525899 36096200

[43] Zheng L, Liu Y, Pan J. Inhibitory effect of pyrvinium pamoate on uveal melanoma cells involves blocking of Wnt/β-catenin pathway. Acta Biochim Biophys Sin (Shanghai). 2017;49(10):890–8. 28981601 10.1093/abbs/gmx089

[44] Rudolf K, Cervinka M, Rudolf E. Dual Inhibition of topoisomerases enhances apoptosis in melanoma cells. Neoplasma. 2010;57(4):316–24. 20429622 10.4149/neo_2010_04_316

[45] Dunsche L, Ivanisenko N, Riemann S, Schindler S, Beissert S, Angeli C et al. A cytosolic mutp53(E285K) variant confers chemoresistance of malignant melanoma. Cell Death Dis. 2023;14(12):831. Available from: https://www.nature.com/articles/s41419-023-06360-4 10.1038/s41419-023-06360-4 PMC10721616 38097548

[46] Meier P, Legrand AJ, Adam D, Silke J. Immunogenic cell death in cancer: targeting necroptosis to induce antitumour immunity. Nat Reviews Cancer Nat Res. 2024;24:299–315. 10.1038/s41568-024-00674-x 38454135

[47] Galluzzi L, Vitale I, Aaronson SA, Abrams JM, Adam D, Agostinis P, et al. Molecular mechanisms of cell death: recommendations of the nomenclature committee on cell death 2018. Cell Death and Differentiation. Nature Publishing Group. 2018;25:486–541. 10.1038/s41418-017-0012-4 PMC5864239 29362479

[48] Amaral T, Sinnberg T, Meier F, Krepler C, Levesque M, Niessner H et al. The mitogen-activated protein kinase pathway in melanoma part I - Activation and primary resistance mechanisms to BRAF inhibition. Eur J Cancer. 2017/02/09. 2017;73:85–92. Available from: https://www.ncbi.nlm.nih.gov/pubmed/28169047 10.1016/j.ejca.2016.12.010 28169047

[49] Amaral T, Sinnberg T, Meier F, Krepler C, Levesque M, Niessner H et al. MAPK pathway in melanoma part II-secondary and adaptive resistance mechanisms to BRAF inhibition. Eur J Cancer. 2017/02/07. 2017;73:93–101. Available from: https://www.ncbi.nlm.nih.gov/pubmed/28162869 10.1016/j.ejca.2016.12.012 28162869

[50] Siroy AE, Davies MA, Lazar AJ. The PI3K-AKT pathway in melanoma. Genetics of melanoma cancer genetics. New York, NY.: Springer; 2016. pp. 165–80.

[51] Carrella D, Manni I, Tumaini B, Dattilo R, Papaccio F, Mutarelli M et al. Computational drugs repositioning identifies inhibitors of oncogenic PI3K/AKT/P70S6K-dependent pathways among FDA-approved compounds. Oncotarget. 2016;7(37). Available from: www.impactjournals.com/oncotarget/10.18632/oncotarget.11318 PMC5312272 27542212

[52] Zheng W, Hu J, Lv Y, Bai B, Shan L, Chen K et al. Pyrvinium pamoate inhibits cell proliferation through ROS-mediated AKT-dependent signaling pathway in colorectal cancer. Med Oncol. 2021;38(2). 10.1007/s12032-021-01472-3 PMC7868320 33554313

[53] Momtazi-Borojeni AA, Abdollahi E, Ghasemi F, Caraglia M, Sahebkar A. The novel role of pyrvinium in cancer therapy. Journal of Cellular Physiology. Wiley-Liss Inc. 2018;233:2871–81. 10.1002/jcp.26006 28500633

[54] Liu R, Chen Y, Liu G, Li C, Song Y, Cao Z, et al. PI3K/AKT pathway as a key link modulates the multidrug resistance of cancers. Cell Death and Disease. Springer Nature; 2020, 11. 10.1038/s41419-020-02998-6 PMC7515865 32973135

[55] Bruno D, Fonseca CG, Proud. Downstream Targets of mTORC1. In: V.A. Polunovsky, P.J. Houghton, editors. Cancer Drug Discovery and Development. Springer Science; 2010. Available from: http://www.springer.com/series/7625

[56] Vultur A, Villanueva J, Krepler C, Rajan G, Chen Q, Xiao M, et al. MEK Inhibition affects STAT3 signaling and invasion in human melanoma cell lines. Oncogene. 2014;33(14):1850–61. 23624919 10.1038/onc.2013.131 PMC3769503

[57] Forschner A, Nanz L, Maczey-Leber Y, Amaral T, Flatz L, Leiter U. Response and outcome of patients with melanoma skin metastases and immune checkpoint Inhibition. Int J Cancer. 2024. 10.1002/ijc.35103 39032035

[58] del Ama LF, Jones M, Walker P, Chapman A, Braun JA, Mohr J, et al. Reprofiling using a zebrafish melanoma model reveals drugs cooperating with targeted therapeutics. Oncotarget. 2016;7(26):40348–61. 27248171 10.18632/oncotarget.9613 PMC5130012

[59] Precazzini F, Pancher M, Gatto P, Tushe A, Adami V, Anelli V, et al. Automated in vivo screen in zebrafish identifies Clotrimazole as targeting a metabolic vulnerability in a melanoma model. Dev Biol. 2020;457(2):215–25. 30998907 10.1016/j.ydbio.2019.04.005

[60] Ciarlo C, Kaufman CK, Kinikoglu B, Michael J, Yang S, D′Amato C et al. A chemical screen in zebrafish embryonic cells establishes that Akt activation is required for neural crest development. Elife. 2017;6. 10.7554/eLife.29145 PMC5599238 28832322

[61] Esumi H, Lu J, Kurashima Y, Hanaoka T. Antitumor activity of pyrvinium pamoate, 6-(dimethylamino)‐2‐[2‐(2,5‐dimethyl‐1‐phenyl‐1 *H* ‐pyrrol‐3‐yl)ethenyl]‐1‐methyl‐quinolinium pamoate salt, showing Preferential cytotoxicity during glucose starvation. Cancer Sci. 2004;95(8):685–90. 15298733 10.1111/j.1349-7006.2004.tb03330.x PMC11159109

[62] Haggerty TJ, Dunn IS, Rose LB, Newton EE, Martin S, Riley JL, et al. Topoisomerase inhibitors modulate expression of melanocytic antigens and enhance T cell recognition of tumor cells. Cancer Immunol Immunother. 2011;60(1):133–44. 21052994 10.1007/s00262-010-0926-x PMC3108190

[63] Gebhardt C, Simon SCS, Weber R, Gries M, Mun DH, Reinhard R et al. Potential therapeutic effect of low-dose Paclitaxel in melanoma patients resistant to immune checkpoint blockade: A pilot study. Cell Immunol. 2021;360. 10.1016/j.cellimm.2020.104274 33383383

