# Supplementary material for: High-throughput drug screening in advanced pre-clinical 3D melanoma models identifies potential first-line therapies for NRAS-mutated melanoma
Source: J Exp Clin Cancer Res. 2025 Oct 1;44:278. doi: 10.1186/s13046-025-03539-9 (PMC12487241; doi:10.1186/s13046-025-03539-9)

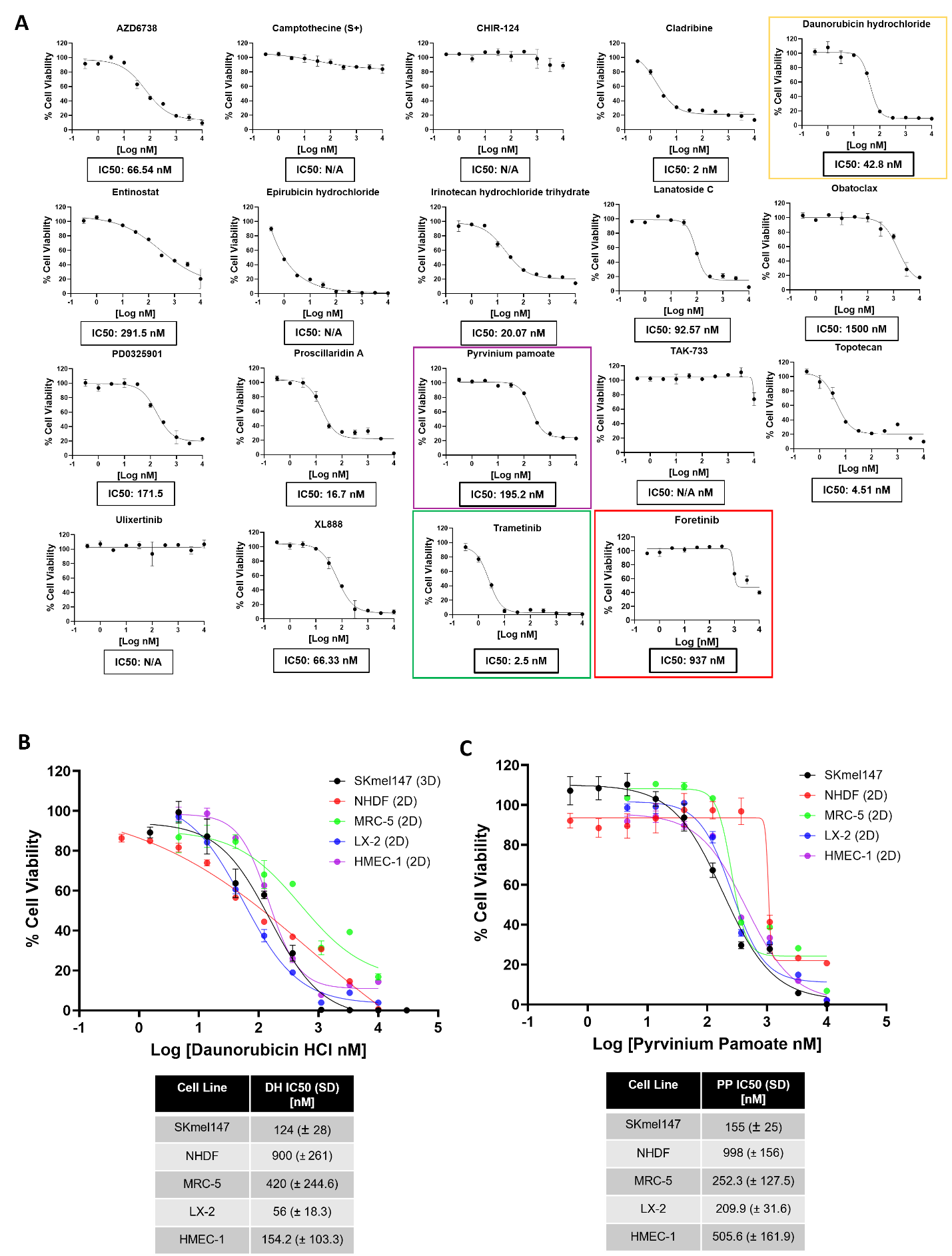


**Supplementary Figure 1.** Validation of selected compounds by DRC generation in SKmel147 spheroids and non-cancer cells. A) DRCs and corresponding IC50 values of hit drugs were generated and calculated by the DSMP. IC50 values are indicated in boxes below the graphs. Trametinib (green) and Foretinib (red) were added as internal assay controls. Daunorubicin HCl is highlighted in yellow and Pyrvinium Pamoate in purple. B-C) DRCs of Daunorubicin HCl (B) and Pyrvinium pamoate (C) tested in non-cancerous cell lines cultured on adherent surface (2D): NHDF skin fibroblasts, MRC-5 lung fibroblasts, LX-2 hepatic stellate cells, and HMEC-1 endothelial cells. CellTiter-Glo® Cell Viability Assay was utilized as readout after 5 days of treatment. Reported IC50 values in the tables are mean±SD of 3 independent biological replicates.


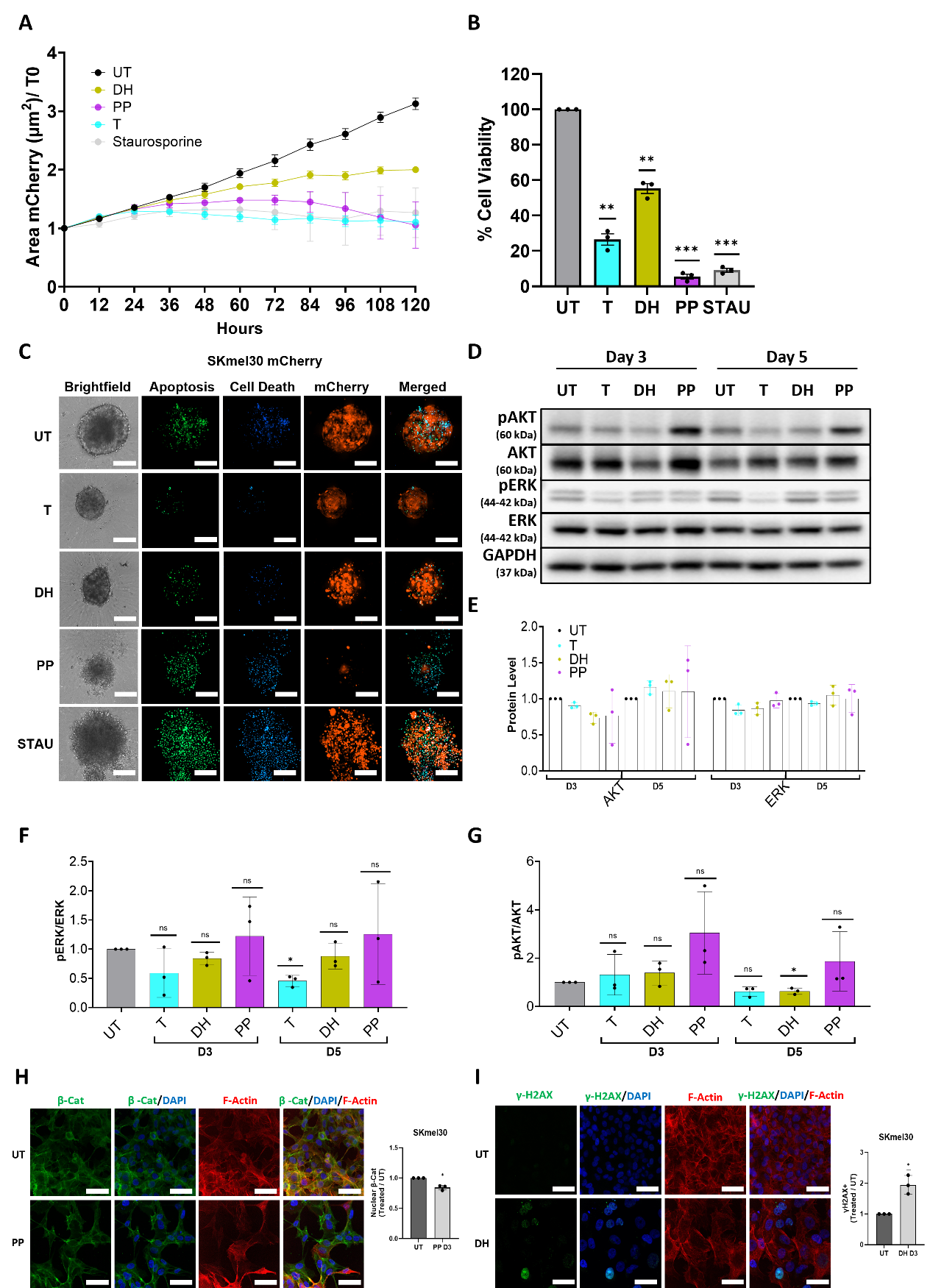


**Supplementary Figure 2.** Daunorubicin HCl and Pyrvinium Pamoate induce inhibition of proliferation, viability and cytotoxic effects in SKmel30 spheroids. A) Proliferation of fluorescent SKmel30-mCherry spheroids treated for 5 days with indicated drugs. Fluorescent images depicting the spheroid area were acquired every 12 hours. (*n*=3, mean±SD). B) Cell viability of SKmel30-mCherry spheroids was assessed after 5 days of drug treatment. Data are normalized to the untreated control. Staurosporine was used as positive control at 400 nM for A and B. (*n*=3, mean±SD); C) Representative pictures of apoptosis and cell death detection in SKmel30-mCherry spheroids after 5 days of treatment. Staurosporine was used as positive control at 1µM. Apoptosis (green) and cell death (blue) were measured upon the addition of the CellEvent Caspase-3/7 and Sytox Blue detection reagents, respectively. Confocal images (20x magnification) of single spheroids are shown. Scale bar = 200 µm (*n*=3*).* D) Western blot of whole cell lysates from SKmel30 spheroids treated for 3 and 5 days with either Daunorubicin HCl (DH), Pyrvinium Pamoate (PP) or Trametinib (T). E) Quantification of the total AKT and ERK protein levels in SKmel30, normalized to GAPDH and to the untreated control. F-G) pERK/ERK and pAKT/AKT ratios respectively in SKmel30 spheroids. GAPDH was used as loading control; data were normalized by and to day-specific untreated controls. Representative blots of three biological replicates are shown. (H) Immunofluorescence (IF) staining of β-Catenin (green), nuclei (DAPI, blue), and F-Actin (Phalloidin, red); Bar plot shows quantification of the relative β-Catenin nuclear signal. (I) IF staining of γH2AX (green), nuclei (DAPI, blue), and F-Actin (Phalloidin, red); Bar plot shows quantification of the relative γH2AX nuclear signal. Cell-line specific IC50 drug concentrations were used for spheroid stimulations in the different assays. UT: untreated, Stau: Staurosporine. One sample T-test was used for B, F, G, H and I for statistical significance testing (**p*≤0.05, ***p*≤0.01, ****p*≤0.001*)*.


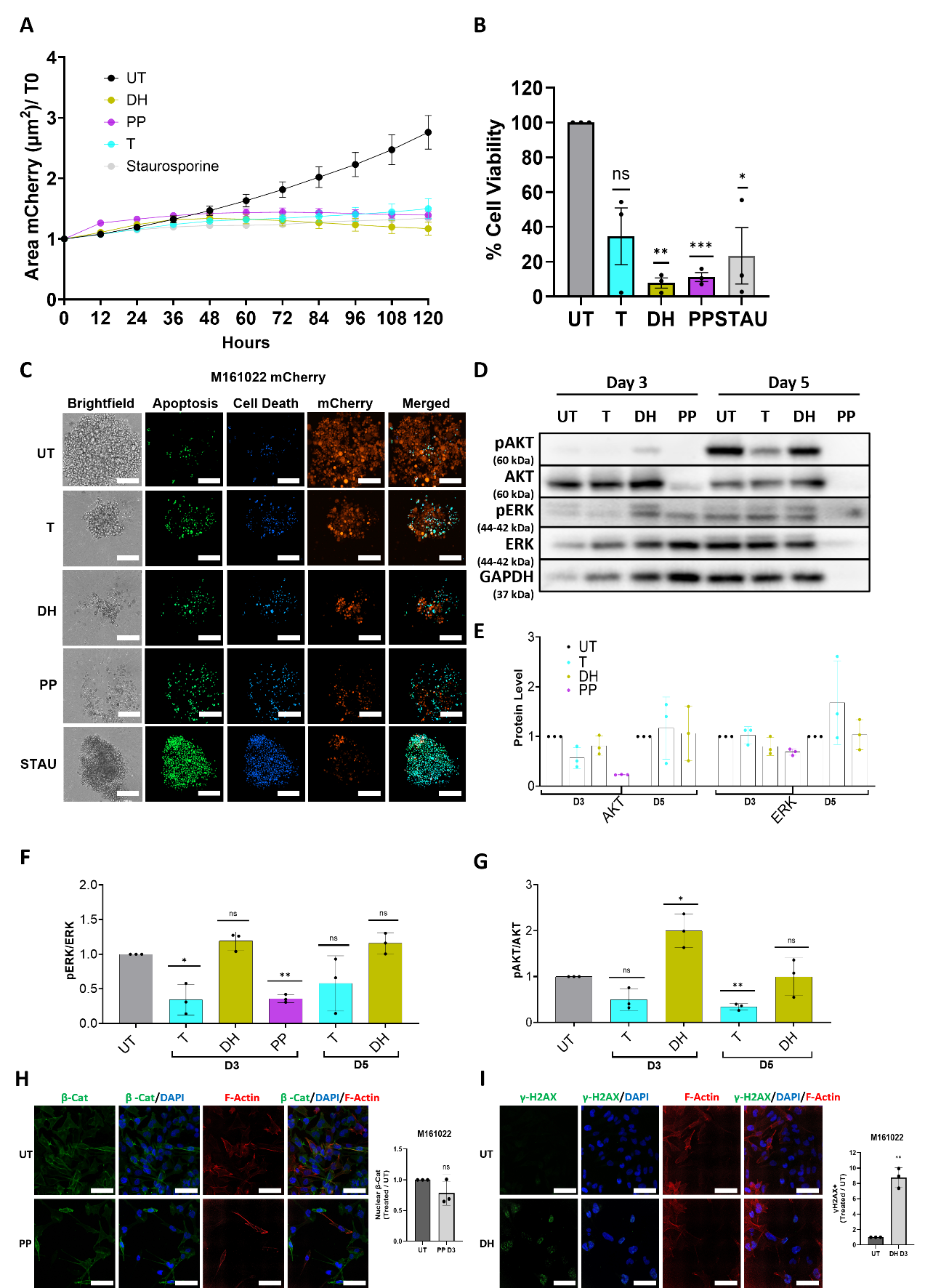


**Supplementary Figure 3.** Daunorubicin HCl and Pyrvinium Pamoate induce inhibition of proliferation, viability and cytotoxic effects in M161022 3D aggregates. A) Proliferation of fluorescent M161022-mCherry 3D aggregates treated for 5 days with indicated drugs. Fluorescent images depicting the 3D aggregates area were acquired every 12 hours (n=3, mean±SD). B) Cell viability of M161022 3D aggregates was assessed after 5 days of drug treatment. Data are normalized to the untreated control. Staurosporine was used as positive control at 200 nM for A and B. (n=3, mean±SD). C) Representative pictures of apoptosis and cell death detection in M161022-mCherry 3D aggregates after 5 days of treatment. Staurosporine was used as positive control at 1µM. Apoptosis (green) and cell death (blue) were measured upon the addition of the CellEvent Caspase-3/7 and Sytox Blue detection reagents, respectively. Confocal images (20x magnification) of single spheroids are shown. Scale bar = 200 µm (n=3). D) Western blot of whole cell lysates from M161022 3D aggregates treated for 3 and 5 days with either Daunorubicin HCl (DH), Pyrvinium Pamoate (PP) or Trametinib (T). E) Quantification of the total AKT and ERK protein levels in M161022, normalized to GAPDH and to untreated control. Day 5 PP treatment was not quantified due to insufficient lysate. F-G) pERK/ERK and pAKT/AKT ratios respectively in M161022 3D aggregates. GAPDH was used as loading control; data were normalized by and to day-specific untreated controls. Representative blots of three biological replicates are shown. (H) Immunofluorescence (IF) staining of β-Catenin (green), nuclei (DAPI, blue), and F-Actin (Phalloidin, red); Bar plot shows quantification of the relative β-Catenin nuclear signal. (I) IF staining of γH2AX (green), nuclei (DAPI, blue), and F-Actin (Phalloidin, red); Bar plot shows quantification of the relative γH2AX nuclear signal. Cell-line specific IC50 drug concentrations were used for spheroid stimulations in the different assays. UT: untreated, Stau: Staurosporine. One sample T-test was used for B, F, G, H and I for statistical significance testing. (ns=not significant, *p≤0.05, **p≤0.01 ***p≤0.001).


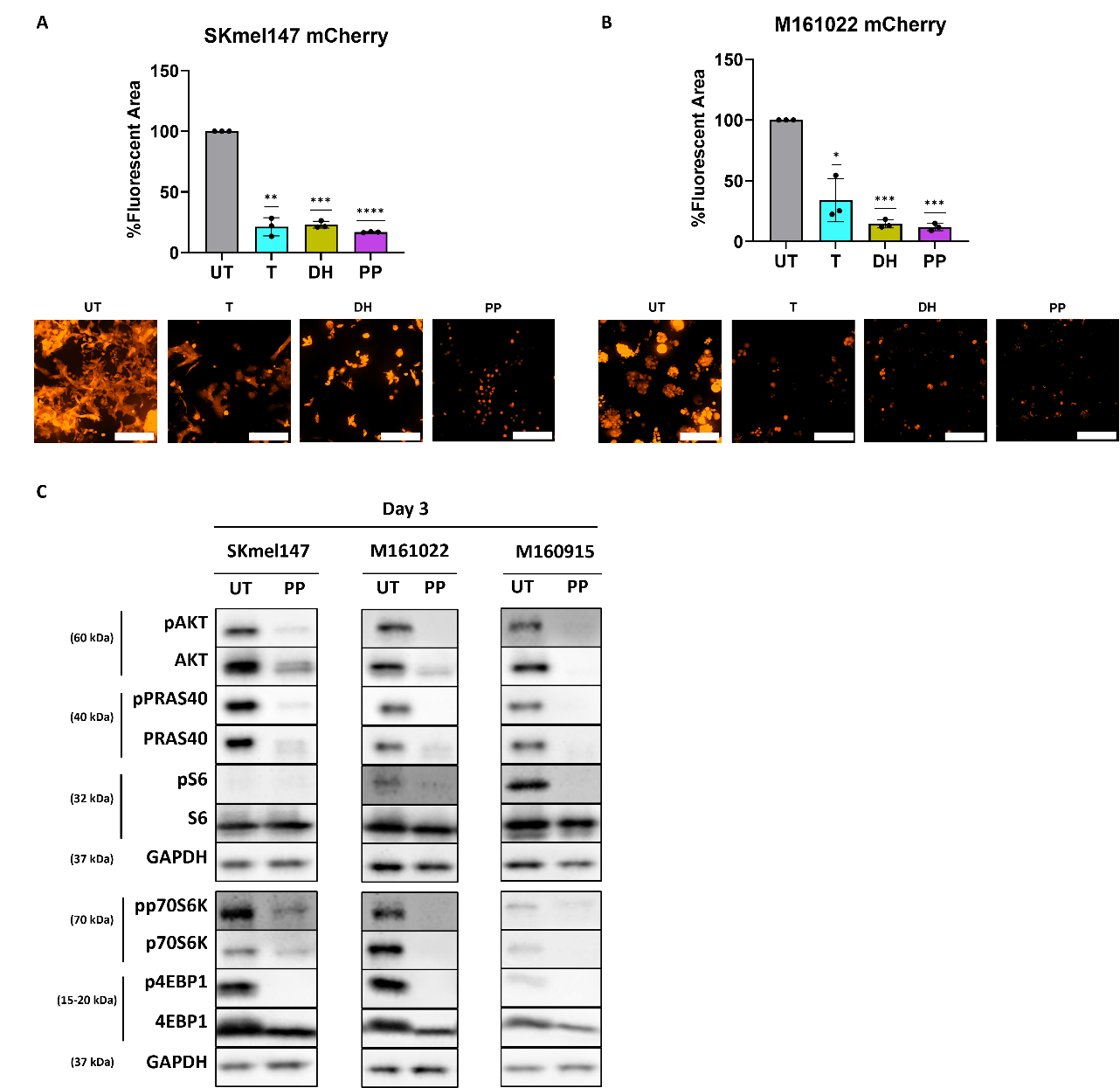


**Supplementary Figure 4.** Hydrogel embedded melanoma cell growth inhibition, and elucidation of the PP mechanism of action. Upper part: plots representing the percentage of fluorescent area of SKmel147-mCherry (A) and the patient-derived cell line M161022-mCherry (B) are showed. Lower part: representative confocal pictures (20x magnification) of hydrogel-embedded SKmel147-mCherry (A) and the patient-derived cell line M161022-mCherry (B) after 5-day treatment are showed. Scale bar = 200 µm. Data are normalized to the untreated control of each specific cell population. One sample T-test was used for A-B for statistical significance testing (n=3. mean±SD; *p≤0.05, **p≤0.01, ***p≤0.001, ****p≤0.0001). C) Western blot of whole cell lysates from SKmel147, M161022, and M160915 spheroids, untreated (UT) and treated for 3 days with Pyrvinium Pamoate (PP). GAPDH was used as loading control. Representative blots of three biological replicates are shown. Melanoma cell line-specific IC50 concentrations of Daunorubicin HCl (DH), Pyrvinium Pamoate (PP) and Trametinib (T) were used.


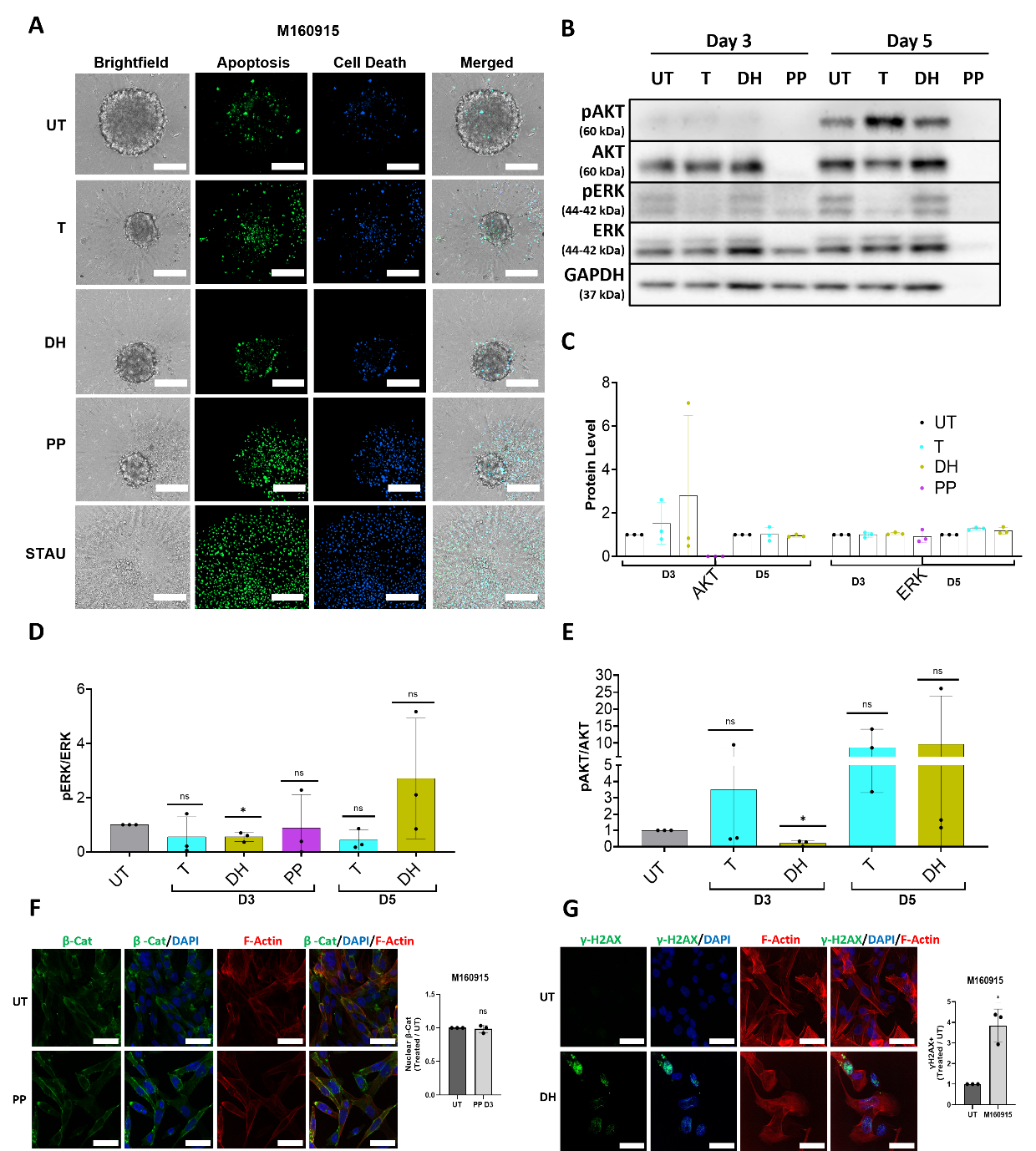


**Supplementary Figure 5.** Daunorubicin HCl and Pyrvinium Pamoate induce cytotoxic effects in M160915 spheroids. A) Representative pictures of apoptosis and cell death detection in M160915 spheroids after 5 days of treatment. Staurosporine was used as positive control at 1µM. Apoptosis (green) and cell death (blue) were measured upon the addition of the CellEvent Caspase-3/7 and Sytox Blue detection reagents, respectively. Confocal images (objective 20x) of single spheroids are shown. Scale bar = 200 µm (n=3). B) Western blot of whole cell lysates from M160915 spheroids treated for 3 and 5 days with either Daunorubicin HCl (DH), Pyrvinium Pamoate (PP) or Trametinib (T) (MEKi). C) Quantification of the total AKT and ERK protein levels in M160915, normalized to GAPDH and to the untreated control. Day 5 PP treatment was not quantified due to insufficient lysate. D-E) pERK/ERK and pAKT/AKT ratios respectively in M160915 spheroids. GAPDH was used as loading control; data were normalized by and to day-specific untreated controls. Representative blots of three biological replicates are shown. (F) Immunofluorescence (IF) staining of β-Catenin (green), nuclei (DAPI, blue), and F-Actin (Phalloidin, red); Bar plot shows quantification of the relative β-Catenin nuclear signal. (G) IF staining of γH2AX (green), nuclei (DAPI, blue), and F-Actin (Phalloidin, red); Bar plot shows quantification of the relative γH2AX nuclear signal. Cell-line specific IC50 drug concentrations were used for spheroid stimulations in the different assays. UT: untreated, Stau: Staurosporine. One sample T-test was used for D, E, F and G for statistical significance testing. (ns=not significant, *p≤0.05).


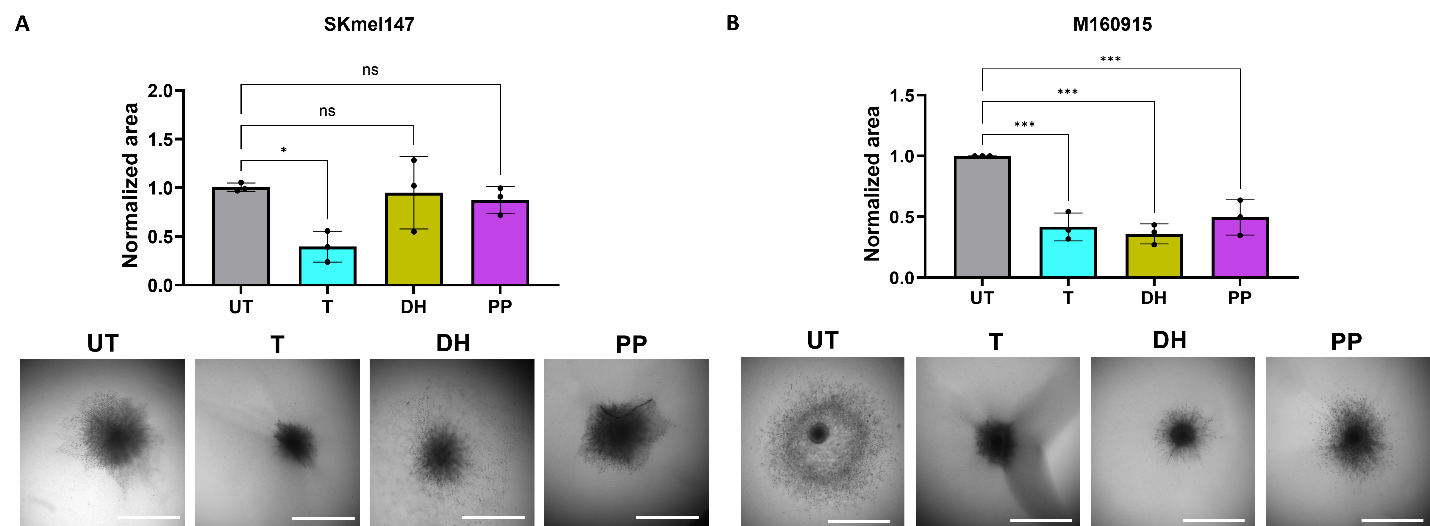


**Supplementary Figure 6.** Inhibitory effect Daunorubicin HCl and Pyrvinium Pamoate on invasion of patient-derived metastatic melanoma spheroids. Bar graphs show the normalized area covered by invasive SKmel147 A) or M160915 B) cells embedded as spheroids in a Collagen I matrix (3D Collagen I assay) after 3 days of treatment. Representative brightfield images (4x magnification) are shown. Scale bar = 1000 µm Ordinary one-way ANOVA was used for A-B for statistical significance testing. (n=3, mean±SD; ns=not significant *p≤0.05, ***p≤0.001).


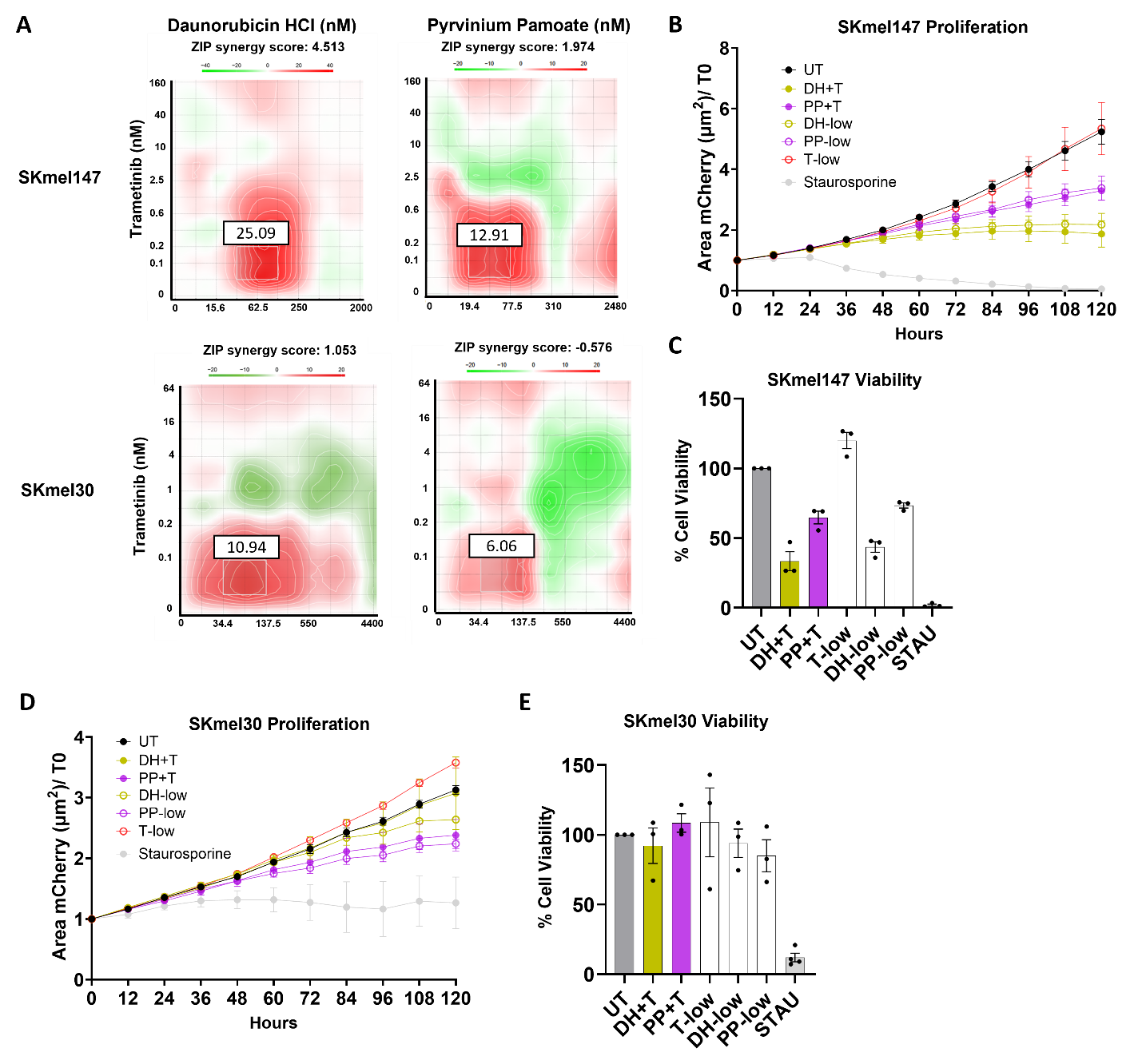


**Supplementary Figure 7.** Combined treatment of Daunorubicin HCl or Pyrvinium Pamoate with Trametinib shows an additive effect. A) Synergy matrices and ZIP synergy scores were generated by the combinatorial treatments of Trametinib plus either Daunorubicin HCl or Pyrvinium Pamoate in SKmel147 and SKmel30 cells cultured as spheroids using the Synergyfinder software. ZIP synergy score values shown above the graphs indicate antagonism (<-10), additive effects (>-10 and <10) or synergism (>10). White boxes delimitate the region with the highest synergistic effects with the relative synergy score above the box. Red areas represent synergistic effects while green areas represent antagonistic effects (n=3). B and D) Kinetic response to drug stimulations of SKmel147-mCherry (B) and SKmel30-mCherry (D) spheroids treated for 5 days with indicated drugs. Fluorescent images of spheroids were acquired every 12 hours and spheroid area was measured. (n=3, mean±SD). C and E) Cell viability of SKmel147 (C) and SKmel30 (E) spheroids was assessed after 5 days of drug treatment. Data are normalized to the untreated control. (n=3, mean±SD). Staurosporine was used as positive control at 200 nM in B and C, and at 400 nM in D and E. DH+T/PP+T= drugs used at synergistic concentrations: T = 0.06 nM and DH/PP = 45 nM. T-low, DH/PP-low = corresponding single drug synergistic concentrations.


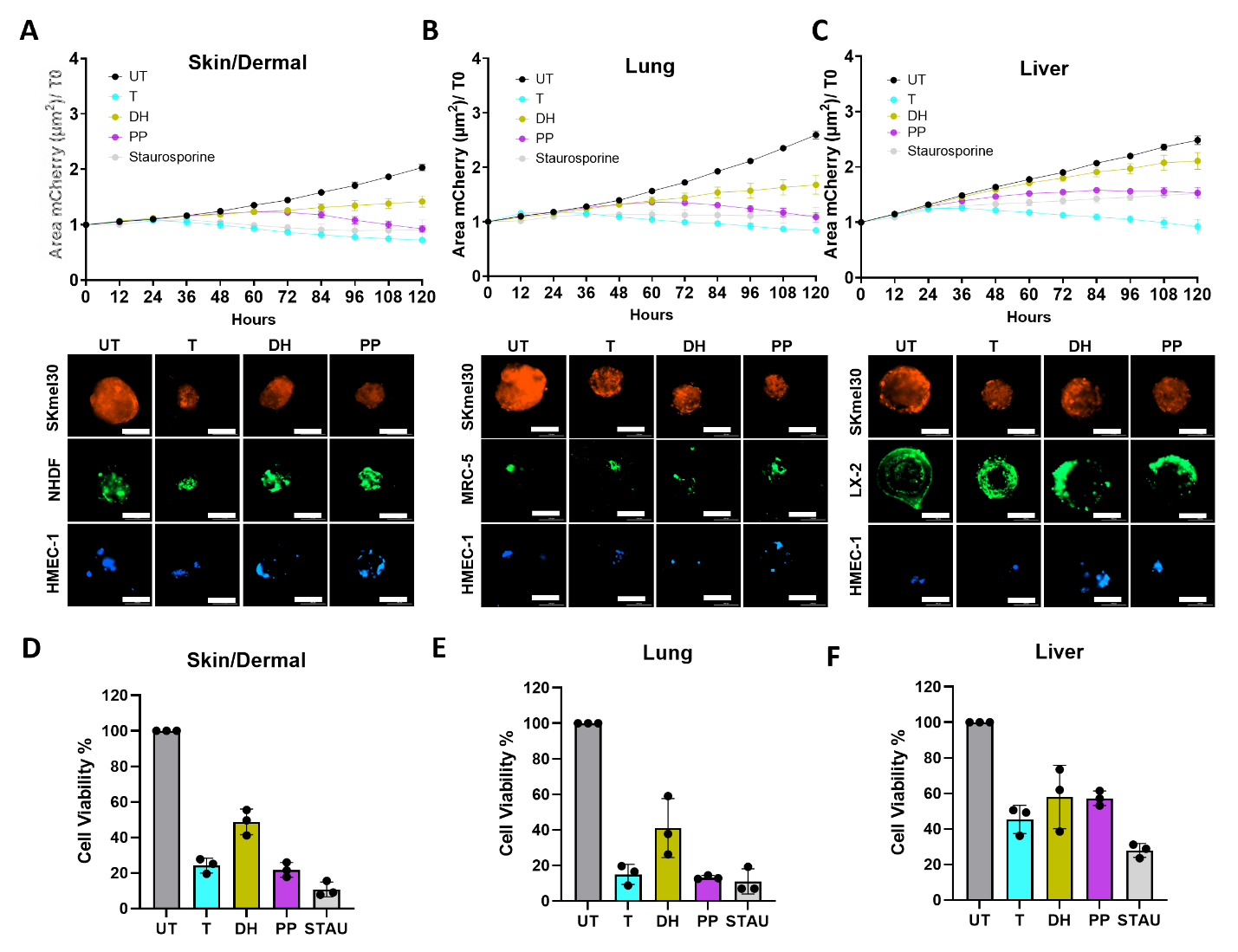


**Supplementary Figure 8.** SKmel30-TME MMS models reveal inhibitory melanoma-specific effect. A-C) Upper panels: Kinetic response of SKmel30-mCherry co-cultures to 5-day drug treatment within 3 different Melanoma Multicomponent Spheroid (MMS) models: “Skin/Dermal” (A), “Lung” (B), and Liver” (C). Images of mCherry fluorescence were acquired every 12 hours and the spheroid area was determined and plotted. Lower panels: confocal pictures (20x magnification) of the different cell populations composing the 3 MMS models after 5 days of drug stimulations: SKmel30-mCherry (red), NHDF/MRC-5/LX-2 (green), HMEC-1 (blue). Scale bar = 200 µm. (n=3. mean±SD) D-F) Cell viability of the three SKmel30-MMS models after 5 days of drug treatment. Data are normalized to the untreated control. Staurosporine was used as positive control at 400 nM. (n=3. mean±SD).


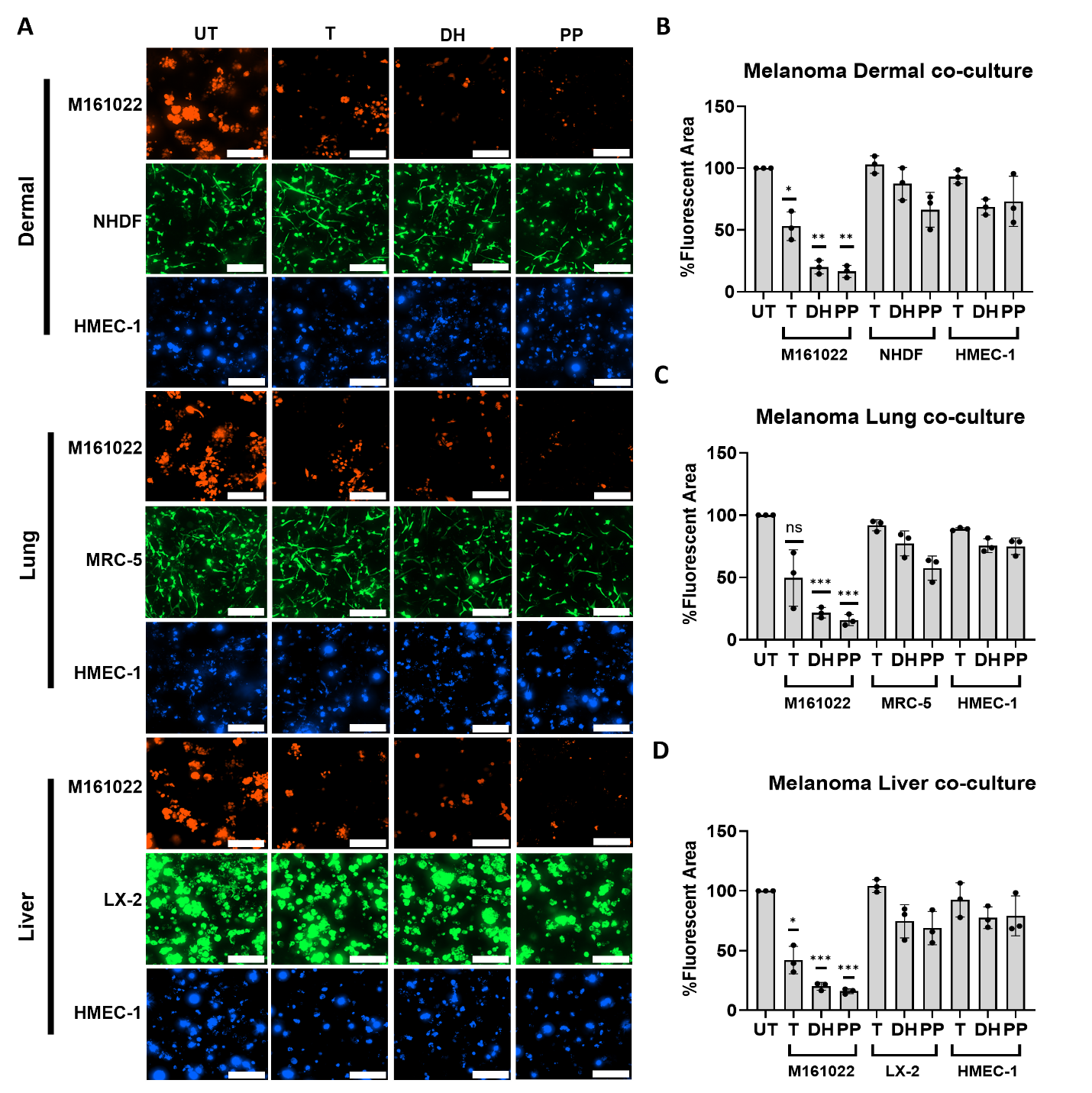


**Supplementary Figure 9.** Daunorubicin HCl and Pyrvinium Pamoate inhibit patient-derived metastatic melanoma cells growth with low toxicity on non-cancerous cells in M161022-TME hydrogel-embedded co-culture models. A) Representative confocal pictures (20x magnification) of 3 hydrogel-embedded M161022-TME co-culture models (“Dermal”, “Lung”, and Liver”) after 5-day treatment: M161022-mCherry (red), NHDF/MRC-5/LX-2 (green), HMEC-1 (blue). Scale bar = 200 µm. B-D) Plots representing the percentage of fluorescent area of the different cell populations in the 3 hydrogel co-culture models as depicted. Data are normalized to the untreated control of each specific cell population. One sample T-test was used for B-D for statistical significance testing. (n=3. mean±SD; ns=not significant, *p≤0.05, **p≤0.01 ***p≤0.001). Melanoma cell line-specific IC50 concentrations of Daunorubicin HCl (DH), Pyrvinium Pamoate (PP) and Trametinib (T) were used.


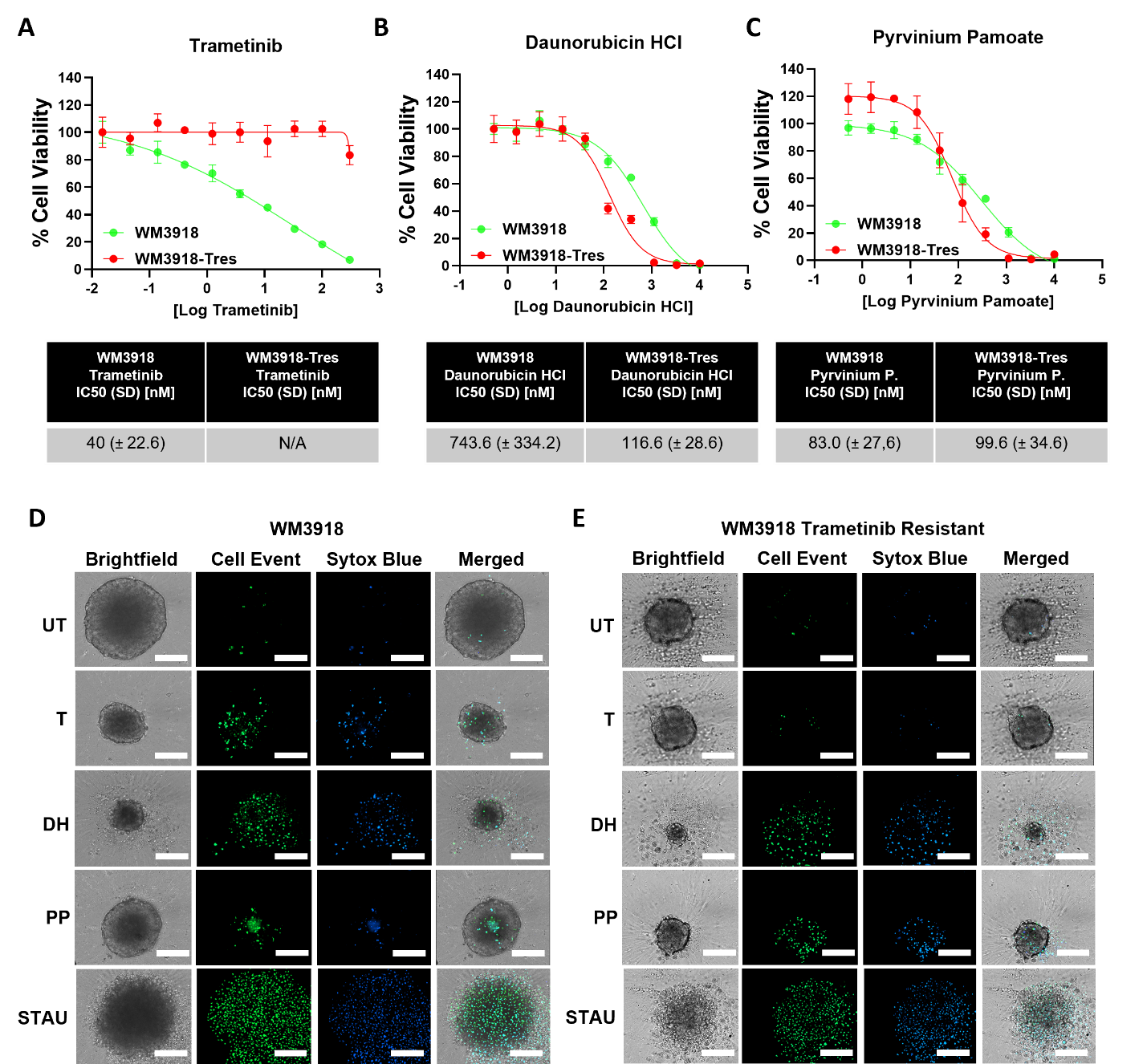


**Supplementary Figure 10.** Daunorubicin HCl and Pyrvinium Pamoate show inhibitory and cytotoxic effects on MEKi-resistant BRAF^wt^/NRAS^wt^ melanoma cell lines. A-C) Representative drug response curves for Trametinib (MEKi) (A), Daunorubicin HCl (B) and Pyrvinium Pamoate (C) in Trametinib-sensitive (green) and -resistant (red) WM3918 cells cultured as spheroids, utilizing CellTiter-Glo® 3D Cell Viability Assay as readout after 5 days of treatment. Reported IC50 values in the tables are mean±SD of 3 independent biological replicates. Tres: Trametinib-resistant. D-E) Representative pictures of apoptosis and cell death detection in WM3918 (D) and T-res WM3918 (E) spheroids that were treated with different compounds for 5 days. Staurosporine was used as positive control at 1µM. Apoptosis (green) and cell death (blue) were measured upon the addition of the CellEvent Caspase-3/7 and Sytox Blue detection reagents, respectively. Confocal images (20x magnification) of single spheroids are shown. Scale bar = 200 µm (n=3).


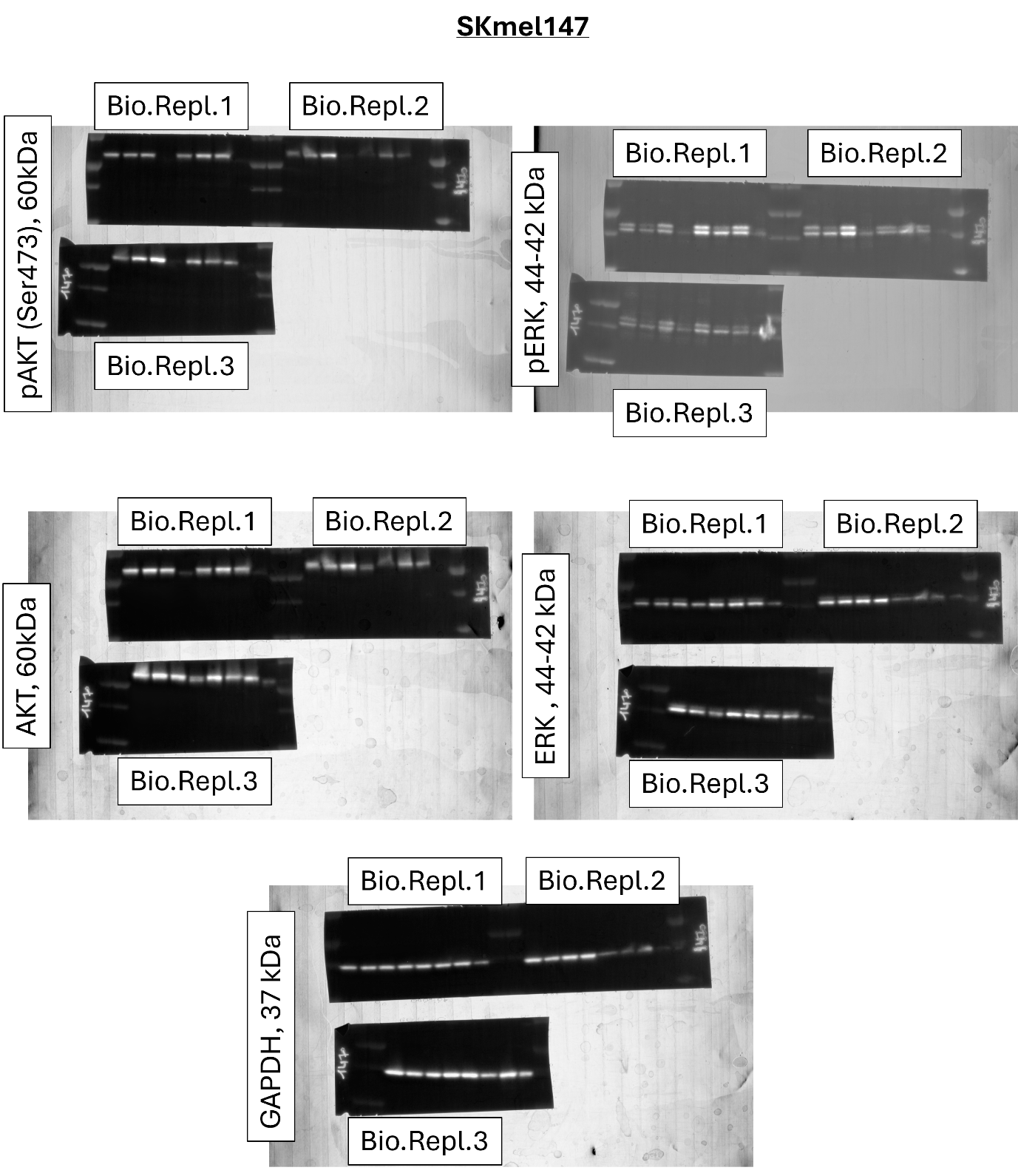


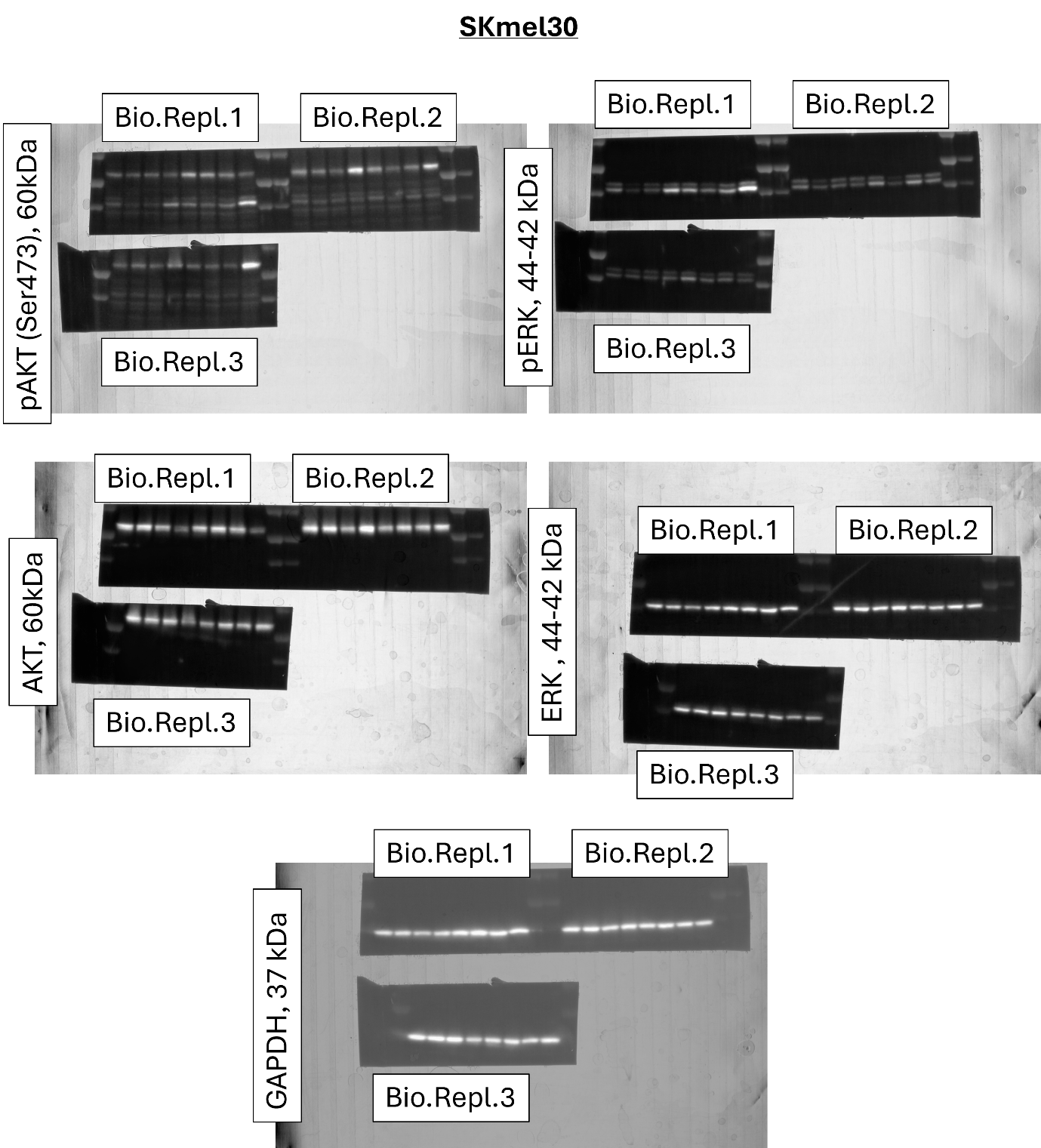


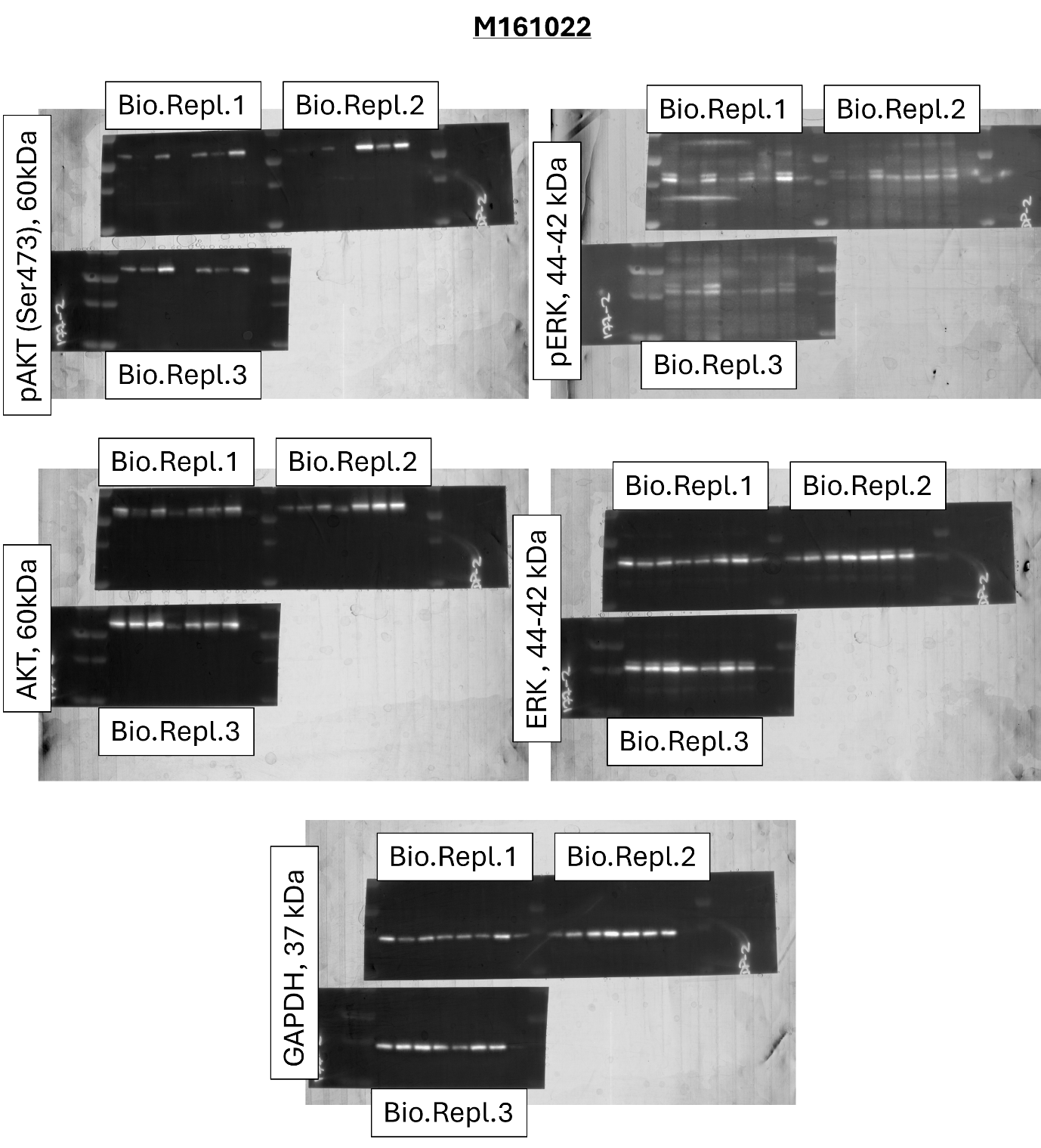


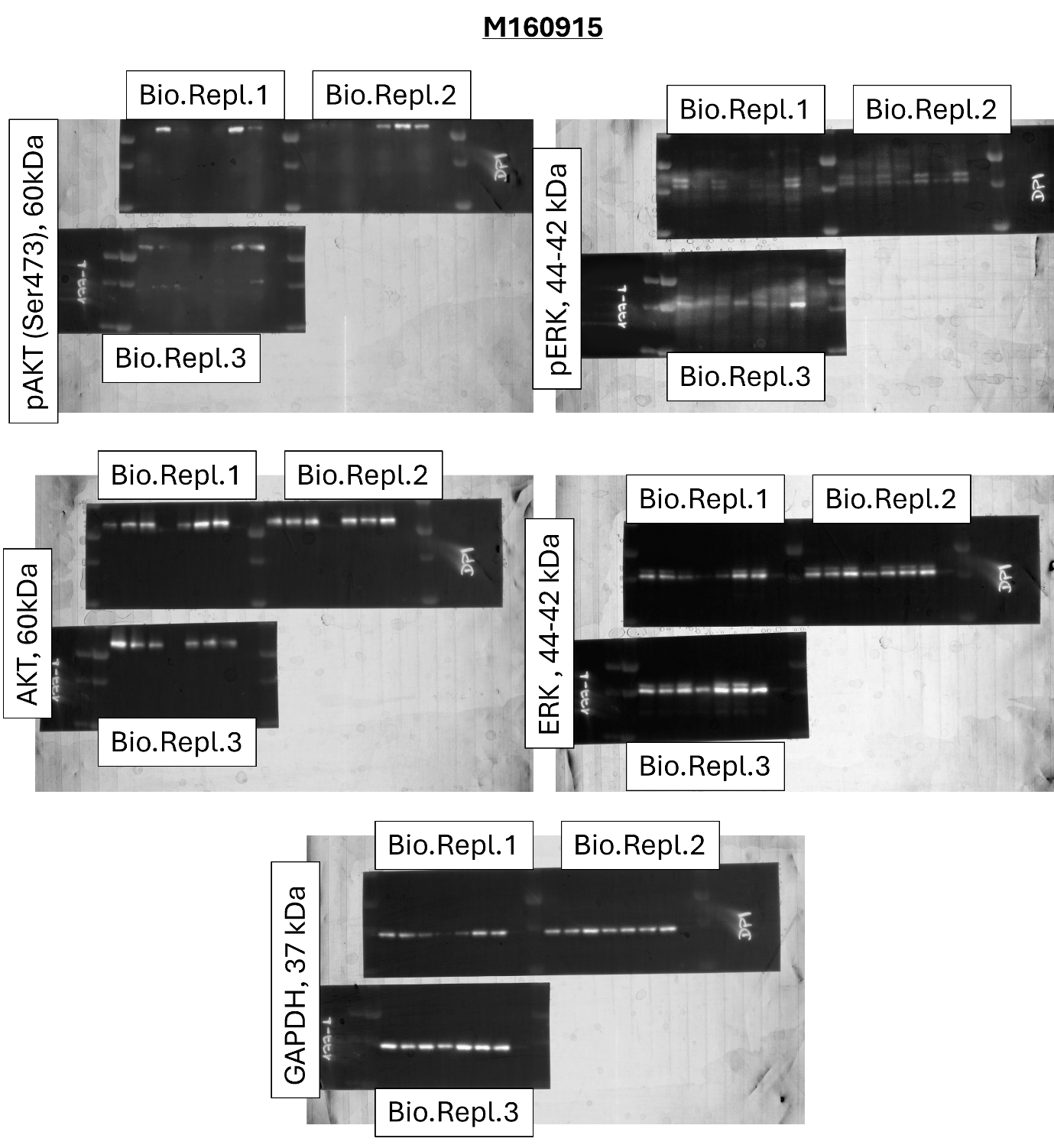


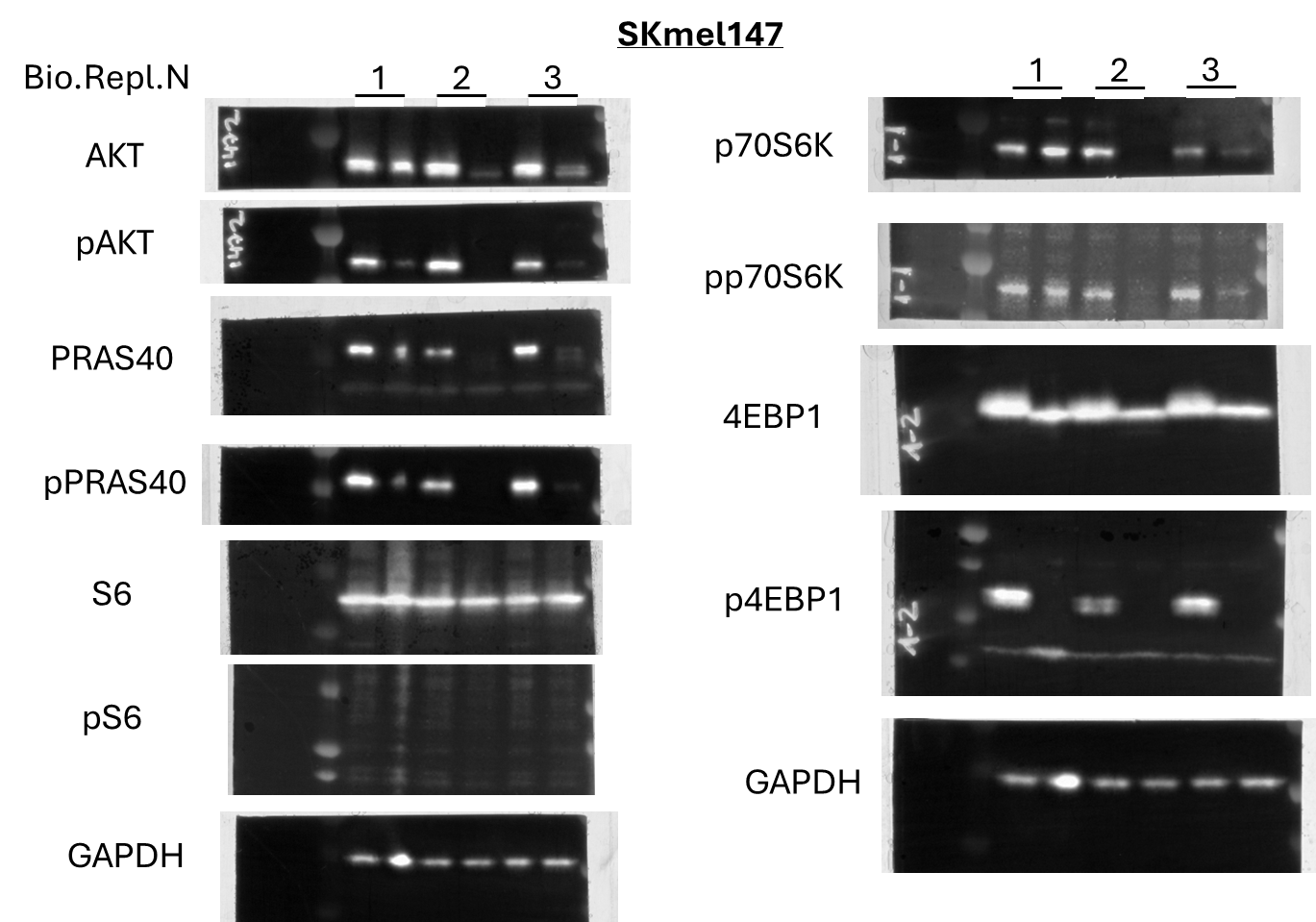


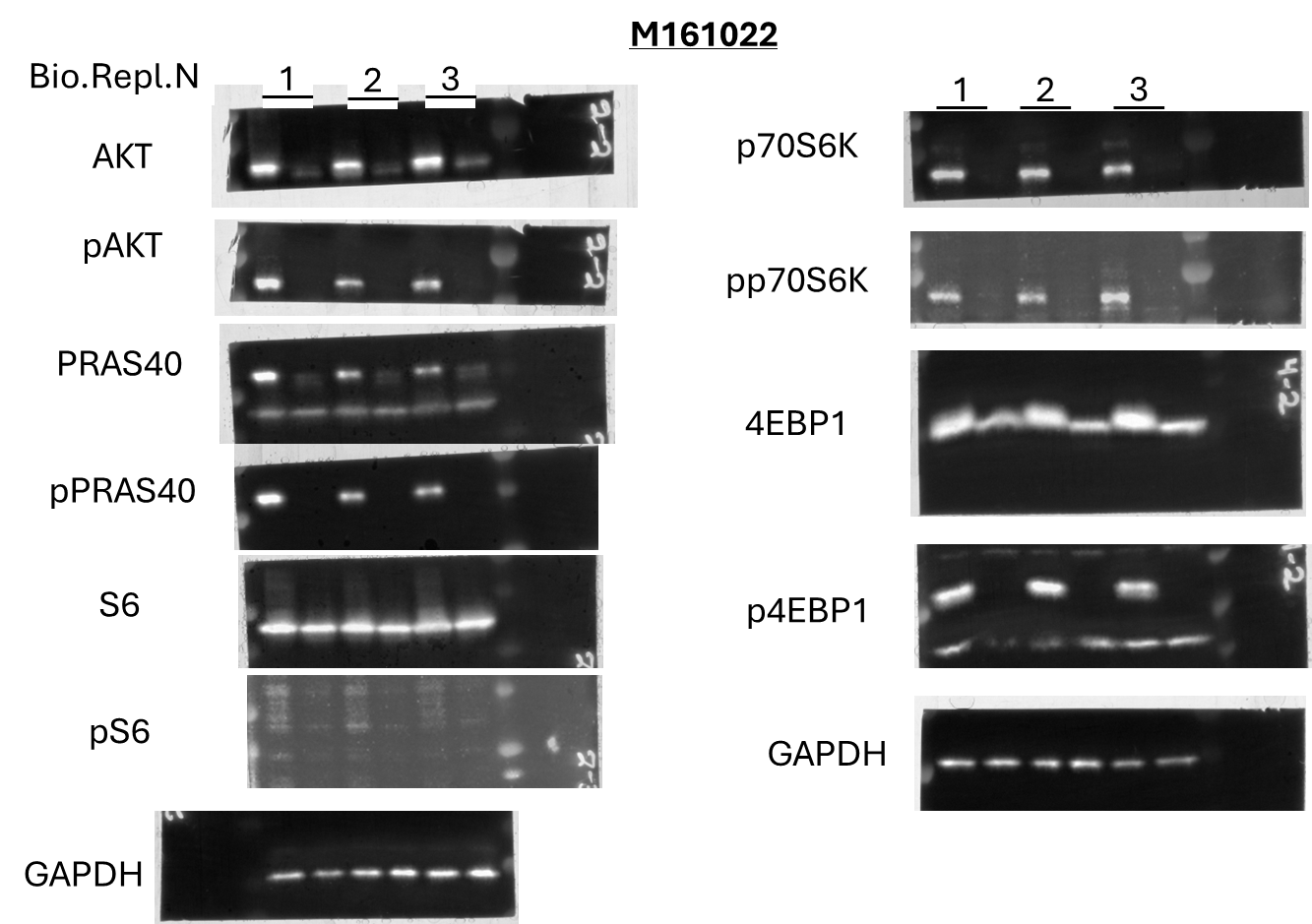


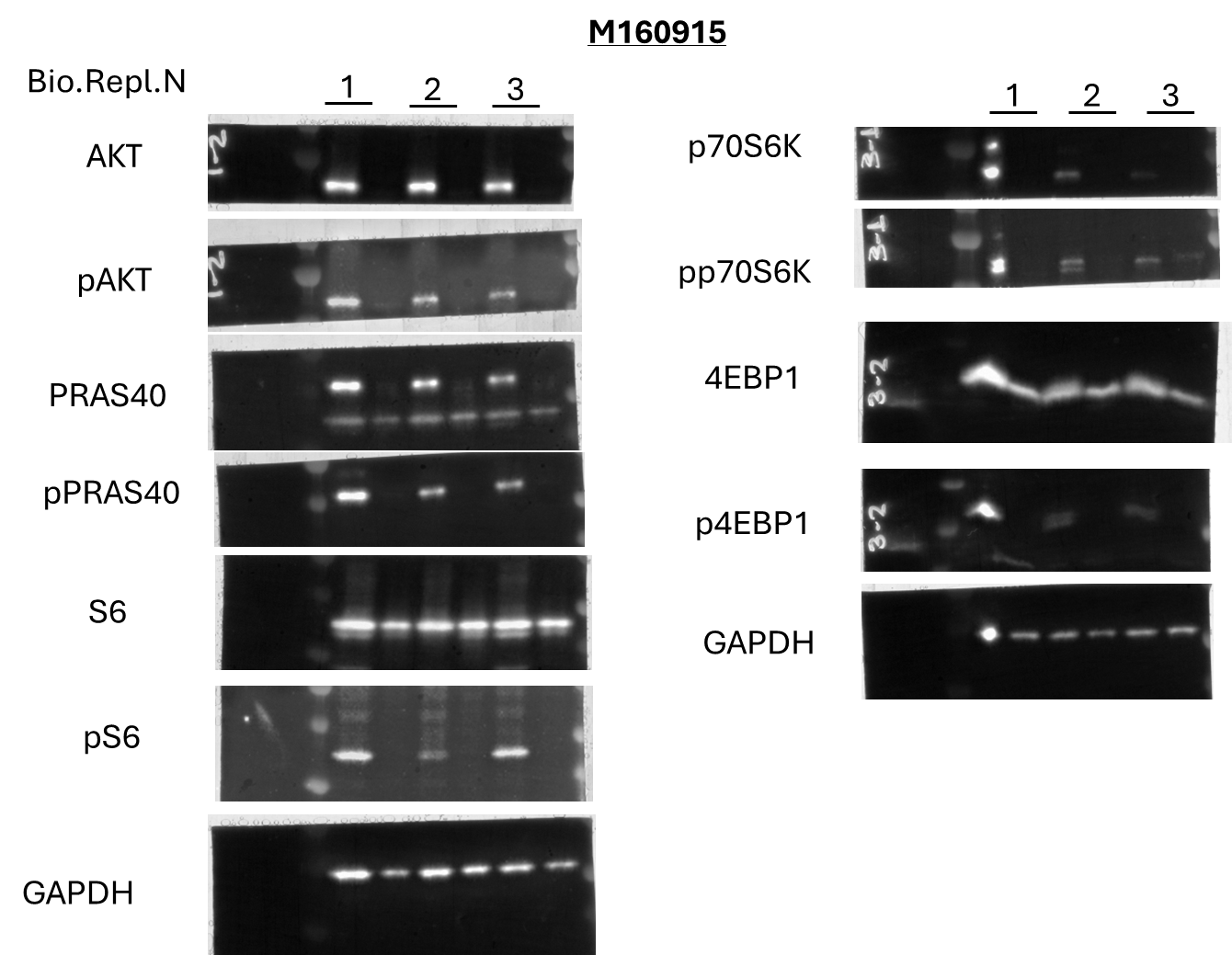

Supplement: Supplementary file 1 — Supplementary Material 1 [file 13046_2025_3539_MOESM1_ESM.docx]
